# Disease- and stage-specific alterations of the oral and fecal microbiota in Alzheimer's disease

**DOI:** 10.1093/pnasnexus/pgad427

**Published:** 2023-12-11

**Authors:** Alba Troci, Sarah Philippen, Philipp Rausch, Julius Rave, Gina Weyland, Katharina Niemann, Katharina Jessen, Lars-Patrick Schmill, Schekeb Aludin, Andre Franke, Daniela Berg, Corinna Bang, Thorsten Bartsch

**Affiliations:** Institute of Clinical Molecular Biology, Kiel University, Kiel, Germany; Department of Neurology, Memory Disorders and Plasticity Group, University Hospital Schleswig-Holstein, Kiel 24105, Germany; Institute of Clinical Molecular Biology, Kiel University, Kiel, Germany; Department of Neurology, Memory Disorders and Plasticity Group, University Hospital Schleswig-Holstein, Kiel 24105, Germany; Department of Neurology, Memory Disorders and Plasticity Group, University Hospital Schleswig-Holstein, Kiel 24105, Germany; Department of Neurology, Memory Disorders and Plasticity Group, University Hospital Schleswig-Holstein, Kiel 24105, Germany; Department of Neurology, Memory Disorders and Plasticity Group, University Hospital Schleswig-Holstein, Kiel 24105, Germany; Department of Radiology and Neuroradiology, University Hospital Schleswig-Holstein, Kiel 24105, Germany; Department of Radiology and Neuroradiology, University Hospital Schleswig-Holstein, Kiel 24105, Germany; Institute of Clinical Molecular Biology, Kiel University, Kiel, Germany; Department of Neurology, Memory Disorders and Plasticity Group, University Hospital Schleswig-Holstein, Kiel 24105, Germany; Institute of Clinical Molecular Biology, Kiel University, Kiel, Germany; Department of Neurology, Memory Disorders and Plasticity Group, University Hospital Schleswig-Holstein, Kiel 24105, Germany

**Keywords:** Alzheimer's disease, microbiome, microbiota, mild cognitive impairment

## Abstract

Microbial communities in the intestinal tract are suggested to impact the ethiopathogenesis of Alzheimer's disease (AD). The human microbiome might modulate neuroinflammatory processes and contribute to neurodegeneration in AD. However, the microbial compositions in patients with AD at different stages of the disease are still not fully characterized. We used 16S rRNA analyses to investigate the oral and fecal microbiota in patients with AD and mild cognitive impairment (MCI; *n* = 84), at-risk individuals (APOE4 carriers; *n* = 17), and healthy controls (*n* = 50) and investigated the relationship of microbial communities and disease-specific markers via multivariate- and network-based approaches. We found a slightly decreased diversity in the fecal microbiota of patients with AD (average Chao1 diversity for AD = 212 [SD = 66]; for controls = 215 [SD = 55]) and identified differences in bacterial abundances including *Bacteroidetes*, *Ruminococcus*, *Sutterella*, and *Porphyromonadaceae*. The diversity in the oral microbiota was increased in patients with AD and at-risk individuals (average Chao1 diversity for AD = 174 [SD = 60], for at-risk group = 195 [SD = 49]). Gram-negative proinflammatory bacteria including *Haemophilus*, *Neisseria*, *Actinobacillus,* and *Porphyromonas* were dominant oral bacteria in patients with AD and MCI and the abundance correlated with the cerebrospinal fluid biomarker. Taken together, we observed a strong shift in the fecal and the oral communities of patients with AD already prominent in prodromal and, in case of the oral microbiota, in at-risk stages. This indicates stage-dependent alterations in oral and fecal microbiota in AD which may contribute to the pathogenesis via a facilitated intestinal and systemic inflammation leading to neuroinflammation and neurodegeneration.

**Significance Statement**

Alterations in the taxonomic pattern of the intestinal microbiome are suggested to impact the pathogenesis of Alzheimer's disease (AD). However, the intestinal microbiome is not fully characterized in different stages of the disease. Our study reveals significant alterations in the oral and fecal microbiota of individuals with AD and mild cognitive impairment, as well as at-risk individuals. The findings suggest a potential link between intestinal microbial communities and neuroinflammation, which may contribute to the pathogenesis of AD. The results provide insight into the microbial compositions of individuals at different stages of the disease and could lead to the development of novel therapeutic strategies targeting the gut–brain axis in AD.

## Introduction

Alzheimer's disease (AD) is a neurodegenerative disorder characterized by progressive cognitive and behavioral impairment leading to dementia (1). The main clinical hallmarks of patients with AD include the development of memory loss, deficits in spatial orientation, attention deficits, emotional disturbance, and personality changes (2). The underlying AD pathology is characterized by the deposition of extracellular amyloid-β (Aβ) plaques and intracellular neurofibrillary tangles composed of hyperphosphorylated tau (tau) protein (3), however, inflammatory processes have been shown to significantly contribute to neurodegeneration (4).

In AD, neurodegenerative processes start decades before clinical symptoms emerge (5, 6), resulting in a preclinical stage of AD. Therefore, early detection of biomarkers and clinical symptoms is essential to identify affected individuals in prodromal, premanifestation, or early disease stages. The most prevalent genetic risk factor for late-onset AD in western population is the APOE4 allele, which encodes a mutated form of the apolipoprotein E (7). In addition to the genetic risk factors, several acquired factors, such as cerebrovascular diseases, diabetes, hypertension, obesity, and other lifestyle diseases increase the risk of developing AD (8–10). In the premanifestation period of the already ongoing neurodegenerative processes, individuals at risk often display a prodromal syndrome defined as mild cognitive impairment (MCI). MCI is characterized by objective cognitive decline without impairment of daily life functionality; however, typical AD biomarkers can already be detected in the cerebrospinal fluid (CSF) (11).

Due to the time course of neurodegenerative processes, environmental factors that may influence disease trajectory early on are of high importance. There is increasing evidence that the human microbiome influences the development and modulation of AD probably via bidirectional communication between the gut and the brain, referred to as the gut–brain axis. Based on experimental research on the microbiome–gut–brain interaction, it is hypothesized that a microbial dysbiosis in the gut may lead to an intestinal barrier dysfunction facilitating intestinal inflammation (12, 13). This intestinal inflammation in turn is thought to result in systemic inflammatory processes which can either indirectly, or directly affect the brain. The indirect pathway includes the release of inflammatory substances (e.g. cytokines and activated immune cells) that can cross the blood–brain barrier and increase its permeability (14, 15). On the other side, bacterial compounds themselves can directly migrate to the brain through the compromised blood–brain barrier (4). In the brain, all these agents promote neuroinflammatory processes, which, in the context of AD, contribute to the neurodegenerative process (4, 13, 16).

The human microbiota is composed of trillions of different microorganisms colonizing the human body, in particular the human gastrointestinal tract (17). Within this complex ecosystem, microbes are involved in key functions for human health including energy extraction, biosynthesis of vitamins, protection against pathogen overgrowth, and education of the immune system (18). Changes in these complex communities have been associated with the development of different inflammatory diseases (19) and in recent years, alterations of the intestinal microbiome were also identified in neurological disorders, such as multiple sclerosis, autism spectrum disorder, Parkinson’s disease, and AD (13, 16, 20, 21).

In the context of AD, studies on the gut microbiome have reported a reduced diversity in patients with AD but differed widely in the kind of disease-associated microbial taxa, in part due to geographically distinct study populations (reviewed in Kowalski and Mulak (22)). Investigations of the oral microbiome of patients with AD, which are strikingly rare, revealed higher species diversity and a higher abundance of biofilm-associated bacteria, like *Porphyromonas* and *Treponema* (23, 24). Notably, *Porphyromonas gingivalis*, an oral pathogen associated with Periodontitis, is frequently found in patients with AD (25–27), and bacterial compounds as lipopolysaccharides (LPSs) have even been detected in the brains of patients with AD (28). The microbiome in prodromal and preclinical stages of AD has so far rarely been investigated. Researchers reported for example higher abundance of *Proteobacteria* and reduced abundance of *Firmicutes* in patients with MCI in contrast to healthy controls (29, 30). Moreover, the APOE4 status appears to have an influence on the gut microbiome in patients with AD as well as Apolipoprotein E (APOE) transgenic mice, particularly regarding butyrate-producing microbes (31).

Although there is increasing evidence of an altered microbial composition in patients with AD that might be related to the pathophysiology of the disease, to date the knowledge about the stage-dependent composition of the fecal and the oral microbiome in the course of AD is sparse. In this study, we performed bacterial 16S ribosomal RNA (rRNA) gene sequencing of the fecal and oral microbiota in individuals with a clinical diagnosis of AD or MCI as well as participants at-risk, as characterized by the presence of an APOE4 allele, in comparison with a cohort of age-matched healthy controls. In addition, we examined the relationship between the microbial composition in the fecal and the oral cavity with disease stages and AD pathology, as reflected by CSF biomarkers, and imaging and clinical cognitive biomarkers.

## Results

### Study design and participant characteristics

Community analyses of the fecal and oral microbiota were performed on fecal samples and oral swabs collected from participants with AD, MCI, at-risk group, and healthy controls. Controls were age and sex matched to AD and MCI participants but not age matched with participants at-risk; therefore, we included age as a covariate in all subsequent analysis. The at-risk cohort was significantly younger than the remaining groups but did not show differences in education or body mass index (BMI). CSF biomarker and cognitive scores were only available for the patient group, indicating no significant differences in biomarker level for patients with MCI and AD, but a worse performance in cognitive testing in patients with AD. Concerning dietary and lifestyle habits, as well as comorbidity prevalence, all groups did not significantly differ (Table 1).

**Table 1.** Participant characteristics.

Characteristics	Controls	At-risk	MCI	AD
	(*n* = 50, fecal = 44, oral = 17)	(*n* = 17, fecal = 17, oral = 8)	(*n* = 17, fecal = 14, oral = 7)	(*n* = 67, fecal = 58, oral = 39)
Age (mean ± SD)	73.82 ± 9.21	63.59 ± 14.3**	75.18 ± 7.6	76.1 ± 7.7
Sex (female, %)	27 (54.0%)	10 (58.82%)	4 (23.53%)	29 (43.28%)
Education (yrs, mean ± SD)	13.17 ± 2.74	13.94 ± 2.44	12.88 ± 3.02	12.57 ± 2.46
BMI (kg/m^2^, mean ± SD)	25.71 ± 6.63	27.96 ± 6.11	25.59 ± 2.36	25.2 ± 3.75
Disease-specific marker				
Disease severity	NA	NA	NA	
Mild (*N*_cases_ (%))				23 (34.33)
Moderate (*N*_cases_ (%))				28 (41.79)
Severe (*N*_cases_ (%))				16 (23.88)
APO E4/3 (*N*_cases_ (%))	0	14 (82.35)	1 (5.88)	0
APO E4/4 (*N*_cases_ (%))	0	3 (17.65%	1 (5.88)	0
Antidementives (*N*_cases_ (%))	0	0	3 (17.65)	6 (8.96)
MoCA *z*-score (mean ± SD)	NA	NA	−1.18 ± 1.68	−2.47 ± 1.34
MMSE *z*-score (mean ± SD)	NA	NA	−1.85 ± 2.1	−2.45 ± 2.28
Cognition score (mean ± SD)	NA	NA	−1.14 ± 1.43	−2.22 ± 1.61^#^
MTA-score (mean ± SD)	NA	NA	1.83 ± 1.0	2.01 ± 0.97
CSF parameters				
p-tau/Aβ_1–42_ (mean ± SD)	NA	NA	0.36 ± 0.87	0.21 ± 0.25
Tau (pg/mL, mean ± SD)	NA	NA	504.29 ± 318.1	658.68 ± 373.75
p-tau (pg/mL, mean ± SD)	NA	NA	199.73 ± 497.38	105.8 ± 144.48
Aβ_1–42_ (pg/mL, mean ± SD)	NA	NA	576.86 ± 233.87	569.35 ± 294.35
Aβ-Ratio_1–40/1–42_ (mean ± SD)	NA	NA	0.62 ± 0.2	0.75 ± 0.11
Blood parameters				
Leukocytes (×10^9^/L, mean ± SD)	NA	NA	5.71 ± 2.73	6.75 ± 2.23
CRP (mg/l, mean ± SD)	NA	NA	2.31 ± 2.41	4.54 ± 8.68
Dietary and lifestyle habits (*N*_cases_ (%))	*N* = 48	*N* = 16	*N* = 11	*N* = 52
Vegetarian	0	0	0	3 (5.77)
Vegan	1 (2.08)	0	0	0
Currently smoking	0	1 (6.25)	1 (9.09)	0
Probiotic consumption	2 (4.17)	2 (12.5)	1 (9.09)	5 (9.62)
Antibiotic consumption (last 6 months)	16 (33.33)	5 (31.25)	1 (9.09)	7 (13.46)*
Gastrointestinal complaints	0	3 (18.75)	0	3 (5.77)
Comorbidities (*N*_cases_ (%))				
Depression	4 (8.33)	1 (6.25)	2 (18.18)	5 (9.62)
Coronary heart disease	7 (14.58)	1 (6.25)	2 (18.18)	10 (19.23)
Diabetes	6 (12.5)	1 (6.25)	2 (18.18)	4 (7.69)
Metabolic syndrome	0	1 (6.25)	0	1 (1.92)
Hypertension	29 (60.52%)	8 (50)	8 (72.73)	31 (59.62)
Inflammatory bowel disease	1 (2.08%)	0	0	2 (3.85)
Hypercholesterolemia	13 (27.08%)	9 (56.25)	2 (18.18)	18 (34.62)

*P < 0.05 and **P < 0.01 compared with controls; ^#^*P* <0.05 compared with MCI group using student's *t*-test or Mann–Whitney *U*-test (in case of violating parametric test requirements) for continuous variables as well as Fisher's exact test in case of categorical data. Unless indicated differently no significant differences were observed between cohorts. MCI, mild cognitive impairment; AD, Alzheimer's disease; SD, standard deviation; BMI, body mass index; APO, apolipoprotein; MMSE, mini-mental state examination; MoCA, Montreal cognitive assessment; CSF, cerebrospinal fluid; p-tau, phospho-tau; Aβ, Amyloid-β; MTA-score, medial temporal atrophy score; CPR, C-reactive protein.

### Composition of the fecal and oral microbiota of healthy controls and AD-related patient groups

In total, 22,510 amplicon sequence variants (ASVs) were identified in the data. After removing ASVs with a total abundance of <50 reads in all samples, the resulting 5,430 ASVs for AD, MCI, at-risk group, and control groups were classified into 185 genera, 76 families, 50 orders, 24 classes, and 11 phyla.

**Fig. 1.**
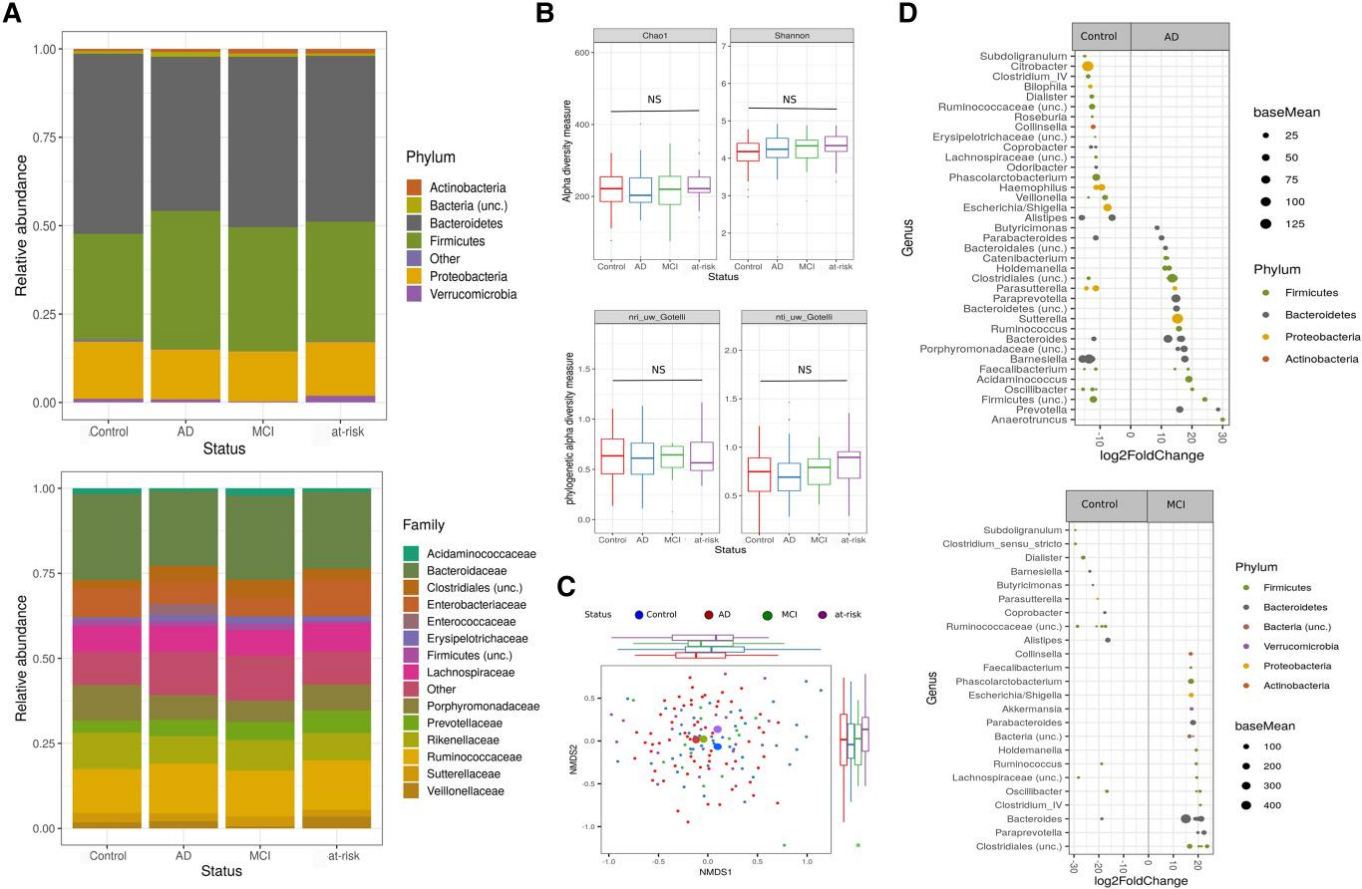
Alteration of fecal microbiota among groups. A) Stacked bar plots of average relative abundance at the phylum and family taxonomic levels. Top 6 phyla (representing 99.6% of all sequence reads) and top 14 families (representing 90.6% of all sequence reads) are shown. B) Alpha diversity measurements (Chao1 and Shannon index) as well as phylogenetic measurements (unweighted NRI and NTI) for patients with AD and MCI and at-risk group versus healthy controls (Chao1 AD = 211, Chao1 control = 215, Chao1 MCI = 219, Chao1 at-risk = 233; Shannon AD = 4.2, Shannon control = 4.1, Shannon MCI = 4.1, Shannon at-risk = 4.3). C) NMDS plot of Bray–Curtis dissimilarities (NMDS, stress value = 0.23) displays sample-wise differences in community composition between health conditions (coloration with respect to group membership, group centroids indicated by large symbols). Marginal boxplots display the grouped distribution of individuals/samples along the respective dimension of the NMDS plot analysis of relative sample ASV composition. D) Differential abundance analysis identified 33 ASVs that were increased and 43 ASVs that were decreased in AD relative to control participants (*P*_FDR_ < 0.05) as well as 24 ASVs increased and 16 ASVs decreased in patients with MCI relative to control participants. Each point represents an ASV. ASVs to the right of the zero line are more abundant and ASVs to the left of the zero line are less abundant in AD and MCI compared with control groups. Abbreviations: MCI, mild cognitive impairment; AD, Alzheimer's disease.

**Fig. 2.**
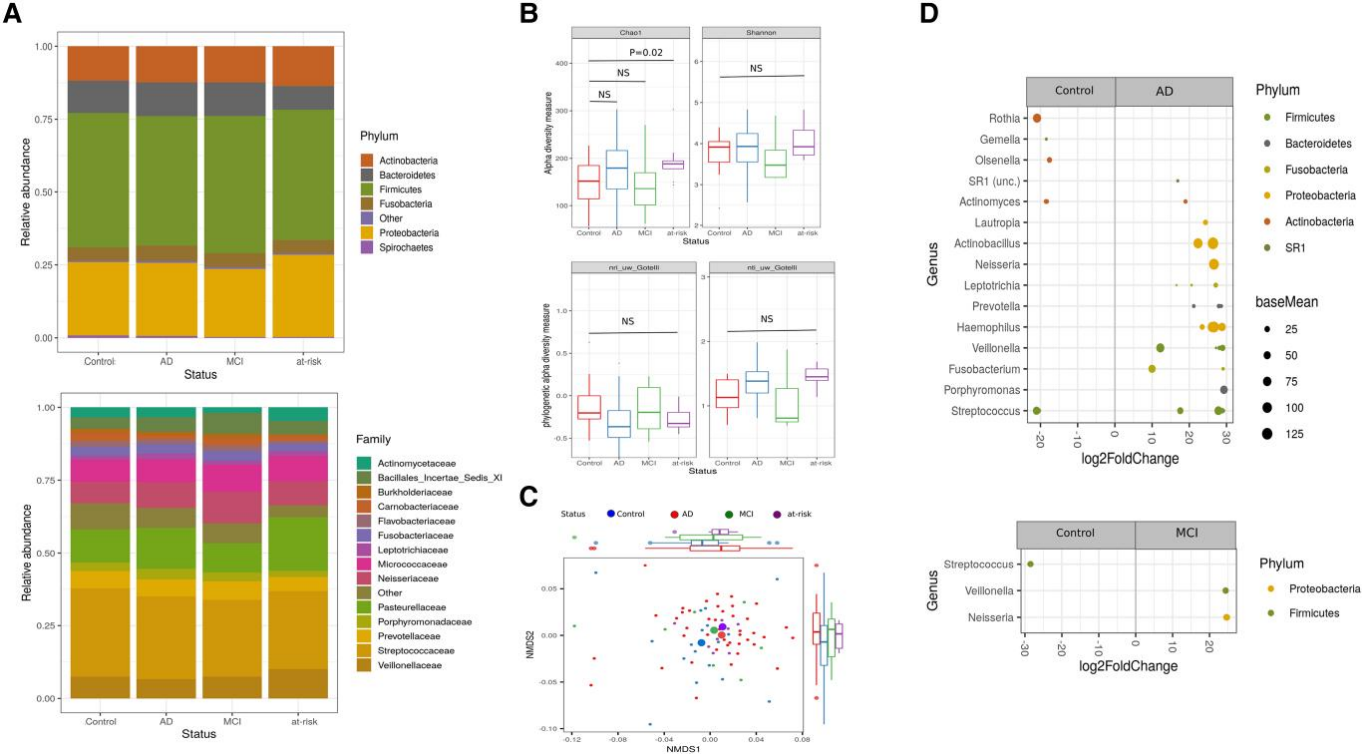
Alteration of oral microbiota in patients with AD. A) Stacked bar plots of average relative abundance at the phylum and family taxonomic levels. Top 6 phyla (representing 99.3% of all sequence reads) and top 14 families (representing 87% of all sequence reads) are shown. B) Alpha diversity measurements for patients with AD and MCI and at-risk group versus healthy controls (Chao1 AD = 174, Chao1 control = 144, Chao1 MCI = 144, Chao1 at-risk = 195; Shannon AD = 3.9, Shannon control = 3.7, Shannon MCI = 3.6, Shannon at-risk = 4.1). C) NMDS plot of Bray–Curtis dissimilarities (NMDS, stress value = 0.22) displays sample-wise differences in community composition between health conditions (coloration with respect to group membership, group centroids indicated by large symbols). Marginal boxplots display the grouped distribution of individuals/samples along the respective dimensions of the NMDS plot. (D) Differential abundance analysis identified 32 ASVs that were increased and 7 ASVs that were decreased in AD relative to control participants (*P*_FDR_ < 0.05) as well as 2 ASVs increased and 1 ASV decreased in patients with MCI relative to control participants. Each point represents an ASV. ASVs to the right of the zero line are more abundant and ASVs to the left of the zero line are less abundant in AD and MCI compared with control groups. MCI, mild cognitive impairment; AD, Alzheimer's disease.

The composition of the fecal microbiota across all 151 participants was dominated by Firmicutes and Bacteroidetes, which made up 47.2 and 34.3% of the total abundance, respectively. Members of the Actinobacteria (0.91%), Verrucomicrobia (1.01%), and Proteobacteria (15%) were the less dominant phyla in the fecal microbiome (Fig. 1A). The oral microbiota was only available for a subset of the donors/patients (*n* = 71), dominated by Firmicutes (45%) and Proteobacteria (26%). Members of the Actinobacteria (12.1%), Bacteroidetes (11.4%), and Fusobacteria (4.8%) were the least abundant bacterial phyla in the community (Fig. 2A).

### AD-associated changes in the fecal and oral microbiota

The composition of the fecal and oral microbiota was characterized using ecological measures including richness and evenness of microbial composition (alpha diversity) and beta-diversity (the similarity or difference in composition between participants). We employed Chao1 species richness (32) and Shannon index (33) to investigate the number of species as well as the evenness of the bacterial communities, while we used the net relatedness index (NRI) and the nearest taxon index (NTI) (34, 35) to evaluate phylogenetic patterns across the whole depth of the phylogenetic community tree or only among closely related species, respectively.

The fecal microbiota of patients with AD was compared with microbiota profiles from controls. Alpha diversity was slightly, but not significantly decreased in patients with AD compared with controls (average Chao1 diversity for AD = 212 [SD = 66]; average Chao1 diversity for controls = 215 [SD = 55]; lm-test *P*_Chao1_ = 0.77; average Shannon diversity for AD = 4.2 [SD = 0.6]; average Shannon diversity for controls = 4.1 [SD = 0.4]; lm-test *P*_Shannon_ = 0.78, *N*_AD_ = 63, *N*_Contr._ = 44; Fig. 1B). However, we observed significant compositional differences in the microbiota between AD and controls (PERMANOVA, Bray–Curtis: *F*_1,105_ = 1.83, *R*^2^ = 0.012, *P* = 0.002; Fig. 1C and Table S1). Differential abundance (DA) analysis of taxa at the ASV level revealed that the fecal microbiota of patients with AD showed significantly altered abundances of 76 ASVs relative to the control group, with 33 ASVs more abundant and 43 ASVs less abundant in AD (Fig. 1C and Table S2). In particular, patients with AD displayed a significantly higher abundance of ASVs belonging to the genera *Bacteroidetes*, *Sutterella*, and *Porphyromonadaceae* and a lower abundance of ASVs in the *Lachnospiraceae*, *Roseburia, Coprobacter,* and *Ruminococcus* in the fecal communities (Fig. 1D and Table S2). Patients with MCI had significantly higher abundances of the genera *Bacteroidetes*, *Parabacteroides*, and *Akkermansia* and lower abundance of *Dialister* and *Butyricimonas* compared with healthy controls. Members of the at-risk group (APOE4+) displayed significantly higher abundances of *Barnesiella* and a lower abundance of *Dialister* and *Oscillibacter* compared with healthy controls.

The oral microbiota of patients with AD was slightly more diverse than in healthy controls, although not significant (average Chao1 diversity for AD = 174 [SD = 60]; average Chao1 diversity for controls = 144 [SD = 50]; lm-test *P*_Chao1_ = 0.08; average Shannon diversity for AD = 3.9 [SD = 0.58]; average Shannon diversity for controls = 3.7 [SD = 0.67]; lm-test *P*_Shannon_ = 0.49, *N*_AD_ = 43, *N*_Contr._ = 17, Fig. 2B). Similarly, no significant compositional differences were observed between the patients with AD group and controls (PERMANOVA, Bray–Curtis: *F*_1,58_ = 0.9380, *R*^2^ = 0.01566, *P* = 0.64, Fig. 2C and Table S1). However, DA analysis revealed 32 ASVs more abundant and 7 ASVs less abundant in AD (Fig. 1C and Table S2). Patients with AD were characterized by a higher abundance of ASVs belonging to the genera *Porphyromonas*, *Fusobacterium*, *Veillonella*, *Actinobacillus*, and *Neisseria*, while *Rothia* and *Gemella* were reduced. Patients with MCI had a higher abundance of ASVs in the genera *Neisseria* and *Veillonella* and lower abundance of *Streptococcus* ASVs compared with healthy controls. Participants from the at-risk group had a higher abundance of *Rothia* and *Prevotella* (Figs. 2A and 2D).

To find taxa that change with disease severity (controls and at-risk = 0, MCI = 1, AD = 2; fecal: *N*(0) = 63, *N*(1) = 15, *N*(2) = 63; oral: *N*(0) = 25, *N*(1) = 7, *N*(2) = 43), we employed trend analyses and identified various taxa nominally significantly associated with disease severity. Although no taxon was significant after correction for multiple testing, we could observe 50 negatively and 43 positively disease severity associated taxa in the fecal microbiome, like, e.g. *Akkermansia* and *Dialister* decreasing with severity and e.g. *Roseburia* increasing (see Fig. S1A and Table S3). In the oral communities, we observed 26 negatively and 30 positively associated taxa. Among others, *Solobacterium uncl.*, *Tannerella forsythia*, and *Leptotrichia buccalis* displayed a positive trend in relation to disease severity, while e.g. *Actinobacillus uncl.*, *Granulicatella elegans*, and *Veillonella* species decrease in the oral communities (see Fig. S1B and Table S3). Overall, these results broadly overlap with the DA analyses, but additionally show an increase in oral periodontal pathogens with disease severity and a decrease in beneficial fecal bacteria like *Akkermansia* with progressing pathology.

**Fig. 3.**
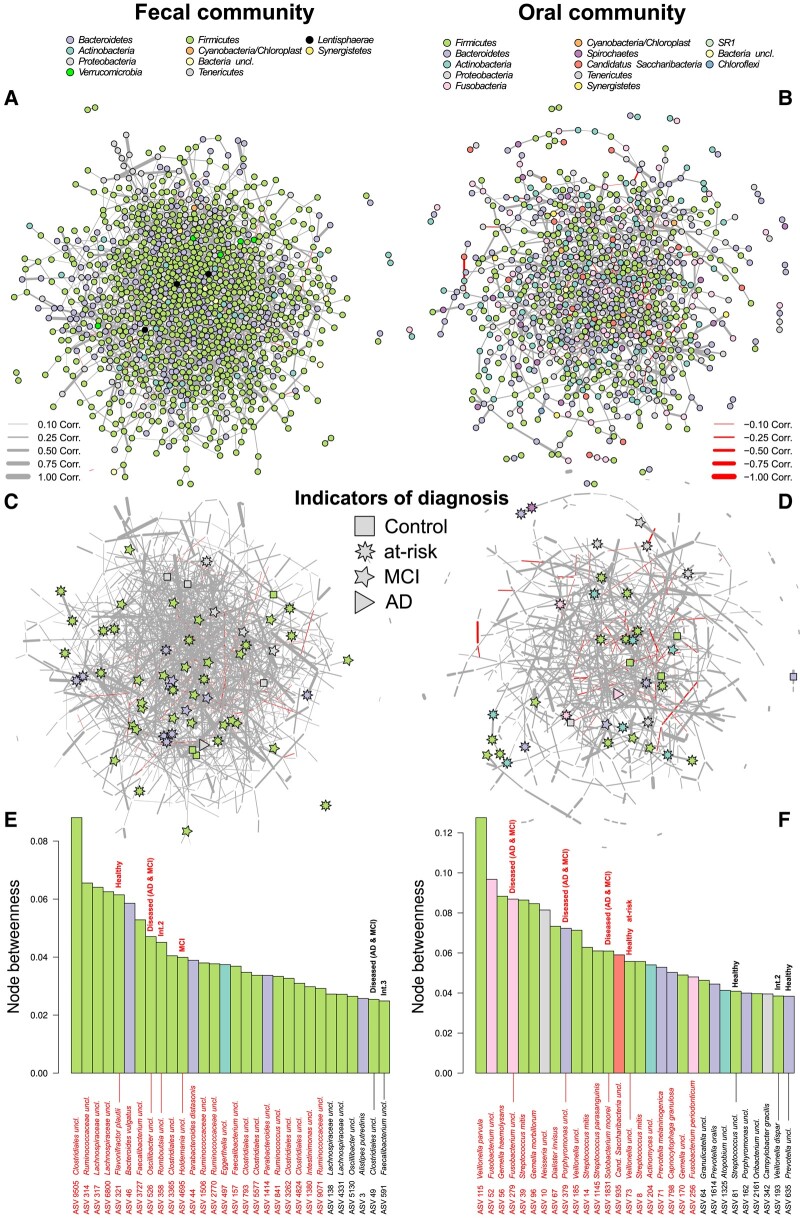
Correlation networks of A) fecal and B) oral microbial communities display positive and negative relationships between ASVs (nodes colored by phylum membership). C) and D) display only the positions of ASVs characteristic for the different health conditions in the either fecal or oral community, as determined by indicator species analysis (36). Network permutation allowed us to assess the significance of network positions/centralities (more central as expected by chance) via one-sided *Z*-tests against the distribution of 10,000 randomized networks (highlighted in red, *P*_FDR_ ≤ 0.05). Bacterial associations to health conditions or disease severity (Int.) were determined by indicator species analyses and marked in the barplots for E) fecal and F) oral communities. Bar colors correspond to phylum membership of the respective ASV. Network layouts are based on the force-directed layout algorithm by Fruchterman and Reingold.

A closer look at the potential bacterial interactions via coabundance network analyses allowed us to identify central bacterial taxa in the fecal and oral microbial communities which may be cornerstones for community and host homeostasis. We identified several fecal bacteria associated with healthy individuals (indicator species analysis: *P*_permuted_ ≤ 0.05; Table S4) or with commonly known probiotic characteristics among the most important taxa in the fecal community network (e.g. ASVs belonging to *Bacteroides*, *Oscillibacter*, *Ruminococcaceae*, and *Faecalibacterium*; Fig. 3A-D). However, mainly members of the Firmicutes appear to be significantly central in the fecal network of which *Romboutsia, Lachnospiraceae*, *Holdemania*, and *Oscillibacter* are highly important in the correlation networks (e.g. node betweenness, node degree, and PageRank index), while also associated to disease characteristics (Tables S4 and S5, Figs. 3E, S2, and S3). Disease-associated bacteria (MCI/disease) play a central role in the oral community network as well. Particularly *Solobacterium moorei* (ASV 1831), *Fusobacterium* uncl. (ASV 279), and *Porphyromonas* uncl. (ASV 379) are opportunistic pathogens of the oral microbiota and characteristic of diseased individuals (Fig. 3F). Interestingly, *Veillonella* (ASV 73) is consistently central and associated with healthy individuals (Tables S4 and S5, Figs. 3F, S2, and S3).

To compare the datasets between the fecal and oral samples included in AD, MCI, and healthy control participants, a Procrustes analysis was performed using the Bray–Curtis distances among fecal and oral samples. The nonmetric multidimensional scaling (NMDS) plots display samples of individuals present in the fecal and oral dataset (Fig. S4) displaying the correlation and rotation of both community topographies. Samples of the same individual are connected by a line and show an overall significant correlation between both community configurations (ASV based: *P* < 0.0010, *m*^2^12 = 0.2038, *r* = 0.8923, *n* = 56, Procrustes). Interestingly, the highest similarity between community topographies (smallest average residuals) was observed in patients with AD (*P*_Contr./AD_ = 0.058, pairwise *t*-test; contr.: 0.0652 ± 0.0148, MCI: 0.0634 ± 0.0127, AD: 0.0562 ± 0.0125; at-risk: 0.0586 ± 0.0108; mean ± SD; see Fig. S4), which translates to more similar community patterns and dynamics in the fecal and oral community among patients with AD. Although community dynamics are correlated between the oral and fecal communities (KEGG Orthology [KO] based: *P* < 0.0010, *m*^2^12 = 0.8397, *r* = 0.4004, *n* = 56; Procrustes), particularly in AD, communities differ strongly in their taxonomic and functional composition (see Fig. S5; Kyoto Encyclopedia of Genes and Genomes).

### Correlation of fecal and oral microbiota with scores of AD pathology

We examined the relationship between the relative abundance of ASVs and levels of CSF biomarkers, general inflammatory blood parameters, neurocognitive assessment, and image scores in all patients with AD and MCI. CSF biomarkers included Aβ_42_ and phosphorylated tau (p-tau). CSF Aβ_42_ is an indicator of amyloid burden, with lower levels in the CSF reflecting greater amyloid deposition in the brain; p-tau is a marker of neurofibrillary tangles, with higher levels reflecting greater neurodegenerative tangle pathology in the brain; the ratio of p-tau/Aβ_42_ incorporates both aspects of pathology, with higher values implying a higher AD severity (10). As a blood marker for general systemic inflammation, we used C-reactive protein (CRP).

We used a general neurocognitive score (called “cognition”) unifying *z*-scores from different screening tools (Montreal cognitive assessment [MoCA], mini-mental state examination [MMSE], and Mattis dementia rating scale [MRDS]) as well as the medial temporal atrophy (MTA) score for evaluating the MTA (ranging from 0 to 4, see Methods).

**Fig. 4.**
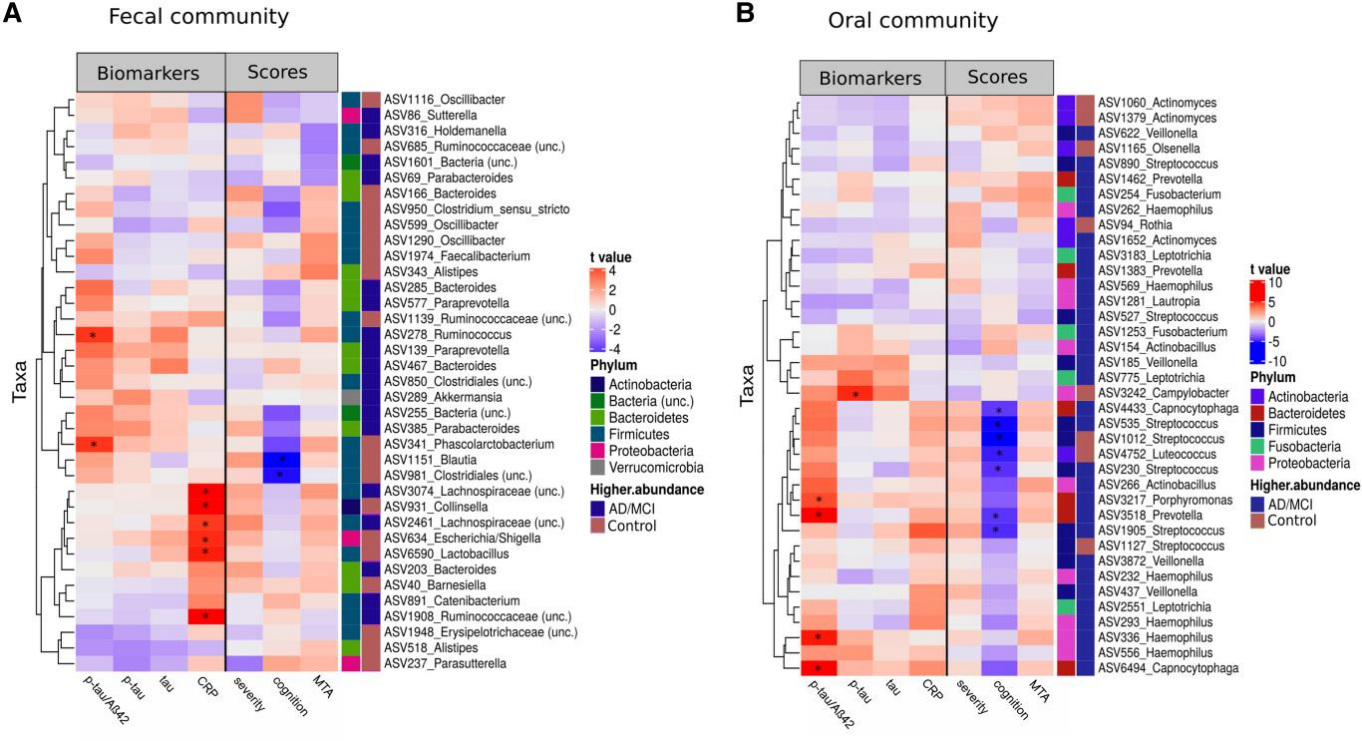
Heatmap of relationships between ASV abundance and biomarkers levels, severity scores, and image scores. A) 37 most strongly correlated ASV from the fecal microbiota (highest *t*-value from linear modeling) were shown. B) 33 most strongly correlated ASVs from the oral microbiota (highest *t*-value from linear modeling) were shown. Asterisks denote significant associations (*P*_FDR_ < 0.05) between taxa and AD scores. Results are from fitting a multiple regression model to the severity measure which included all covariates (disease status, age, and sex), in addition to each single taxon (centered log ratio transformed [CLR]). Red color inside the heatmap (darker cells in the Biomarker columns) indicates a positive correlation between scores and ASV abundance and the blue color (darker cells in the Scores columns) indicates a negative correlation between scores and ASV abundance. Color bars on the right of each plot indicate phylum for each ASV and the group (AD/MCI or controls) in which each ASV is more abundant. MCI, mild cognitive impairment; AD, Alzheimer's disease, CRP, C-reactive protein; MTA, mediotemporal atrophy score; p-tau, phospho-tau; Aβ_1–42_, Amyloid-β_1–42_.

Fecal and oral microbiota diversity and single taxa of the oral and fecal microbiota correlated in several instances with CSF biomarkers, neurocognitive *z*-scores, and the MTA score (Table S6). Linear regression tests revealed several significant associations between scores and single taxa, after adjusting for confounding factors, like age, and sex. The fecal microbiota in particular, showed a positive relationship of ASVs belonging to *Ruminococcus* and *Pascolacubacterium* with CSF p-tau/Aβ42 while members of the *Lachnospiraceae*, *Collinsella*, and *Escherichia/Shigella* were positively associated with CRP levels. In addition, an increased abundance of ASVs belonging to *Blautia* and *Clostridiales* was associated with lower values of the neurocognitive severity score cognition (Fig. 4A).

Among genera that were more abundant in patients with AD and MCI (*Bacteroides*, *Ruminococcus*, *Butyricimonas*, *Paraprevotella*, and *Sutterella*), we observed a positive relationship between ASV abundance and AD and MCI pathology (increasing amyloid burden), as implied by positive correlations (*t*-value >0) with CSF p-tau/Aβ_42_ ratios. In contrast, ASVs and genera that are less abundant in patients with AD and MCI (e.g. *Alistipes, Parasutterella, Barnesiella uncl.*, and *Erysipelotrichaceae*), displayed a negative relationship (*t*-value <0) to AD and MCI pathology (decreasing amyloid burden, CSF p-tau/Aβ_42_; Fig. 4A).

Oral bacteria displayed significant positive associations (*t*-value >0) between ASV abundances, particularly *Porphyromonas*, *Haemophilus*, and *Capnocytophaga*, and CSF p-tau/Aβ_42_ (increasing amyloid burden), as well as positive associations of ASVs belonging to the genus *Campylobacter* and CSF p-tau (increasing neurofibrillary tangles, Fig. 4B). In addition, the abundances of several ASVs belonging to the genera *Streptococcus* and *Capnocytophaga* were negatively associated (*t*-value <0) with the neurocognitive score, which implies influences of the microbial communities on regulatory cognitive processes in the central nervous system.

Among AD- and MCI-associated oral bacteria (*Haemophilus*, *Veillonella*, *Prevotella*, and *Actinobacillus*), we observed a positive relationship between bacterial abundance and increasing AD and MCI pathology, which was indicated by positive correlations between bacterial abundance and CSF p-tau/Aβ_42_ (*t*-value >0). Among bacteria that are less abundant in patients with AD and MCI (i.e. *Actinomyces* and *Streptococcus*), we observed a negative relationship between bacterial abundance and AD pathology (*t*-value <0, Fig. 4B).

### Microbial functional differences present between healthy individuals and patients with AD

To understand the functional consequences of AD and MCI on the microbiota, we imputed functions based on ASV abundances via PiCRUSt2. By investigating the imputed functional spectra, we identified significant, community-wide functional differences between healthy and diseased fecal communities (healthy [controls, at-risk]/diseased [MCI, AD]: *F*_1,139_ = 4.8695, *P* = 0.0073, *R*^2^ = 0.0339, Bray–Curtis distance, *N*_Contr._ = 44, *N*_AD_ = 63, *N*_MCI_ = 15, *N*_at-risk_ = 19, PERMANOVA), which also carried down to differences between the different pathological states (controls/AD/MCI/at-risk: *F*_3,137_ = 2.2744, *P* = 0.0294, *R*^2^ = 0.04744, Bray–Curtis distance, PERMANOVA). However, the strongest differences were present between healthy individuals and patients with AD (*F*_1,105_ = 6.3991, *P* = 0.0022, *R*^2^ = 0.0574, Bray–Curtis distance, PERMANOVA; Fig. S6 and Table S7). In the smaller oral sample cohort, we found no systematic functional community differences with respect to the underlying health conditions and diagnoses (controls/AD/MCI/at-risk: *F*_3,71_ = 0.9880, *P* = 0.4192, *R*^2^ = 0.0401, healthy (controls, at-risk)/diseased (MCI, AD): *F*_1,73_ = 0.1918, *P* = 0.9928, *R*^2^ = 0.0026, Bray–Curtis distance, *N*_Contr._ = 17, *N*_AD_ = 43, *N*_MCI_ = 7, *N*_at-risk_ = 8, PERMANOVA; Fig. S6 and Table S7).

Interestingly, investigating the communities for individual functional differences, we identified several genes and pathways differentially abundant among individuals (oral functions: 248, fecal functions: 129, *P*_FDR_ ≤ 0,01; see Fig. S6, Tables S8 and S9), while differentially abundant functions can only be detected in oral samples when healthy individuals (controls and at-risk) are contrasted with diseased patients (AD and MCI; oral: 328, fecal: 0, *P*_FDR_ ≤ 0.01, Table S10). Enrichment analysis, focusing on Kyoto Encyclopedia of Genes and Genomes (KEGG) pathways further revealed differential functional representation between healthy and diseased communities. We observed significant enrichment of functions involved in amino acid metabolism in healthy oral communities (e.g. “Arginine and proline metabolism” and “Phenylalanine metabolism”), while diseased communities displayed a consistent overrepresentation of functions involved in “Sulfur metabolism” and “Taurine and hypotaurine metabolism” (*P*_FDR_ ≤ 0.05, Table S11). Fecal communities did not show many overlapping differential enrichments among health conditions. Interestingly, among other differentially abundant functions, we detected components of the Curli amyloid protein to be more abundant in orla community of healthy controls compared with patients with AD, as well as healthy vs. diseased individuals, like *csgB* (“minor curlin subunit”), *csgE* (“curli production assembly/transport component”), *csgF* (“curli production assembly/transport component”), and *csgG* (“curli production assembly/transport component CsgG”; Tables S9 and S10). This protein assemblage plays an important role in biofilm formation, and its role in inflammatory processes is currently debated (37).

## Discussion

Microbiota in the human gastrointestinal tract are suggested to impact the development and progression of AD (38, 39). Here, we studied the relationship between AD, MCI, and high-risk genotypes in the oral and fecal microbiota.

We found a distinct microbial composition of the fecal and the oral microbiota from patients (AD and MCI) to age-matched controls as well as to participants at-risk. We further demonstrated that levels of differentially abundant genera correlate with CSF biomarkers of AD pathology and showed that the community patterns and dynamics in the fecal and oral community are more similar among patients with AD in contrast to healthy controls.

### Alterations in fecal microbiota in AD and MCI

In general, the slightly decreased diversity in the fecal microbiome parallels results from other studies investigating alterations in fecal microbiome in patients with AD (29, 40). Although the majority of the studies in patients with AD report a decreased diversity in the fecal microbiome (reviewed in Kowalski and Mulak (22)), the differently abundant genera widely differ. In this study, we observed a slightly decreased abundance of the phylum Bacteroidetes in patients with AD which is not only in accordance with previously reported findings (41) but also conflicts with other reports (29, 40, 42). However, increased abundances were detected in several genera within Bacteroidetes, including the genus *Bacteroides* which were also more abundant in MCI in comparison with the control group and the at-risk group (see Fig. S7). An increased abundance of *Bacteroides* has previously been reported in patients with AD (40).

Within the Bacteroidetes phylum, we further observed a significantly increased abundance of unclassified genus *Porphyromonadaceae* in patients with AD in contrast to healthy controls and in contrast to the at-risk group. *Porphyromonadaceae* are gram-negative bacteria and are enriched in the feces of patients with colorectal cancer, liver diseases (43), and cirrhosis, a disease associated with cognitive impairment due to hepatic encephalopathy (44). Interestingly, the abundance of *Porphyromonadacea* seems to correlate with a lower cognitive level, even unrelated to the diagnosis of cirrhosis (45). Here, the higher abundance of this species in patients with cognitive decline suggests that *Porphyromonadaceae* might indeed be involved in the modulation of cognitive performance, though this has to be confirmed in future studies.

In addition, we observed a general decreased abundance of the phylum Proteobacteria in patients with AD, while we detected an increased abundance of the genus *Sutterella* (Proteobacteria). *Sutterella* has been shown to be drastically increased in patients with autism spectrum disorder (46), which is associated with AD (47), and also shares pathophysiological hallmarks (48, 49).

Most of the species within the phyla Bacteroidetes and Proteobacteria are gram-negative and contain proinflammatory LPSs in their outer membrane promoting inflammatory processes (50, 51). *Porphyromonas gingivalis* LPS for example drives inflammation through activation of Toll-like receptors and cytokines (52, 53). Higher circulating levels of LPS have been associated with increased Aβ deposition in elderly patients with cognitive complaints (54) and were found to colocalize amyloid and tau deposition in the brain of patients with AD (55). This indicates a higher abundance of intestinal gram-negative bacteria to potentially promote/facilitate neuroinflammatory processes due to direct interaction with the pathological proteins Aβ and tau in the brain of patients with AD.

Furthermore, abundance of the phylum Firmicutes was increased in patients with AD. However, within Firmicutes, a reduction was seen in the family *Lachnospiraceae* and the genus *Roseburia*, which all harbor species that produce the metabolite butyrate, and their decrease have been previously associated with AD (42, 56) and also Parkinson (57) although the general role of microbiota-derived short-chain fatty acids (SCFAs) in AD is discussed controversially (58). Besides, *Ruminococcus* was increased in feces of patients with AD of the present study and the higher abundance was associated with low neurocognitive performance. *Ruminococcus* was suggested to predict lower levels of brain N-acetylaspartate, a biochemical indicator of neuronal health, and is decreased in neuron metabolic disturbance (57).

The correlative analysis revealed that genera identified as more abundant in AD were associated with greater AD pathology while genera identified as less abundant in AD were associated with less AD pathology. In particular, we observed associations in patients with AD between higher abundance of *Bacteroides, Ruminococcus, Butyricimonas*, and *Acidaminococcus* with a greater AD pathology indicated by a higher CSF p-tau/Aβ_1–42_ ratio. On the other hand, *Lachnospiraceae* and *Veillonella* showed strong correlations in healthy participants, with greater abundance of these bacteria associated with less AD pathology suggesting these bacterial taxa may be protective against development or progression of AD pathology. Although the differentially abundant taxa differ between studies, associations with differentially abundant taxa and AD pathology measured by CSF biomarker have previously been shown (40).

Coabundance network analyses allowed us to identify central bacterial taxa in the fecal and oral microbial communities which may be important for community and host homeostasis. We identified several fecal bacteria associated with healthy controls, belonging among others to the genera *Bacteroides, Akkermansia*, and *Oscillibacter/Ruminococcaceae*. *Akkermansia muciniphila* or its components appear to show a universal importance for host well-being and its reduction, as shown in DA and trend analyses, has already been reported for several diseases (55, 59). However, mainly members of the *Firmicutes* appear to be significantly central in the community networks, including several SCFA producers like *Faecalibacterium* (60) or other potentially beneficial bacteria (e.g. *Parabacteroides*) (61). In particular, ASVs belonging to *Romboutsia*, *Lachnospiraceae*, and *Oscillibacter* are highly important/influential in the correlation networks in general, while also associated with disease characteristics. Thus, members of these bacteria might play important roles in driving pathological dynamics in the fecal communities.

### Alterations in oral microbiota in AD and MCI

Whereas the stool microbiota has gained much interest in chronic neuroimmune diseases during the last years, a growing attention has been given to the oral microbiota. The oral microbiota is considered to play a key role in the development and progression of chronic inflammatory dental diseases such as Periodontitis (62). Studies have shown an association between periodontal diseases and AD, possibly facilitated by the oral microbiota (24). The oral microbiota may be able to promote neuroinflammation via direct effects, like migration of bacterial substances (e.g. LPS or bacterial amyloid) to the brain, or via indirect effects like compromising the blood–brain barrier which promotes the passage of inflammatory agents (24).

In this study, we found an increased alpha diversity in the oral microbiota of patients with AD, in parallel with previous findings (23, 26, 27). In detail, we found a significantly higher abundance of species involved in periodontal disease among patients with AD or correlated with disease severity, including gram-negative bacteria within the *Porphyromonas* genus, *Leptotrichia* genus, and *Tannerella* (member of “red complex”), whose higher abundance has been previously reported to be associated with AD (26, 27). In particular, *P. gingivalis* is dominantly involved in the pathogenesis of periodontitis (24, 62) and epidemiological studies have proven a relationship to AD (25). Indicating a potential direct interaction of *P. gingivalis* with the AD pathology, postmortem studies revealed the presence of *P. gingivalis* LPS as well as *P. gingivalis* DNA and protease gingipain in the brains of patients with AD but not in control patients (28, 63). On a mechanistic level, lipid A structural modifications of *P. gingivalis* LPS have been shown to induce inflammation via Toll-like receptor pathways as well as expression of cytokines (52, 53).

Furthermore, we observed a significantly higher abundance of members of the *Proteobacteria*, especially *Haemophilus*, *Neisseria*, and *Actinobacillus* (Actinobacteria) in oral samples of patients with AD and MCI. Whereas all of these species are known to be part of the oral microbial flora (64), the higher proportion of those gram-negative bacteria in patients with AD is noteworthy. All of those genera have related species causing severe diseases in humans (65). Particularly, a significant increase in abundance of *Haemophilus* genus has been observed in a previous study in patients with Multiple Sclerosis a neuroinflammatory disease (66).

Moreover, correlative analysis revealed that the increased abundance of *Haemophilus*, *Porphyromonas*, *Prevotella*, and *Capnocytophaga* was associated with a greater AD pathology measured by CSF p-tau/Aβ_1–42_ ratio, whereas the higher abundance of *Rothia*, *Olsenella*, and *Actinomyces* found in healthy controls were associated with less AD pathology. However, additional data on the oral microbiota of patients with AD in general are necessary to understand the overall role of these bacteria in disease manifestation and progression.

Network analyses revealed *Solobacterium moorei*, *Fusobacterium*, and *Porphyromonas* to be opportunistic pathogens of the oral microbiota in highly central and important network positions. *Solobacterium moorei* (originally known as *Bulleidia moorei*) is a main elicitor of haltosis due to its capacity to convert cystein to H_2_S (67). Interestingly *S. moorei* appears to be only capable to produce volatile sulfur compounds from mucin proteins in the presence of an exogenous protease (e.g. Arg-gingipain produced by *P. gingivalis*) (68)⁠⁠, while *Porphyromonas* uncl. and *Fusobacterium nucleatum* (two potential pathogens) display direct positive correlations with *S. moorei* in the oral community network (see Fig. S8).

In accordance with what we found in the fecal microbiota, gram-negative bacteria were dominant in the oral microbiota of patients with AD and MCI and even associated with a higher burden of pathological Aβ and tau. These findings suggest that gram-negative periodontal pathogens or their components (e.g. LPS) may interact directly with pathological processes, particularly facilitating neuroinflammation as part of the neurodegenerative cascade in AD (4). However, the role of Aβ in the context of microbial interaction is discussed controversially as it has been shown to be bactericidal and its deposition and accumulation as a response to bacterial invasion could be misguided (69).

### Disease stage–dependent alterations of microbiota

In contrast to the disease cohorts, the at-risk group did not show significant changes in alpha or beta-diversity of the fecal microbiota, but within the phylum Bacteroidetes, we could identify the genus *Paraprevotella* and *Barnesiella* to be more abundant in APOE4 carriers in contrast to healthy controls. The intestinal microbiome in the APOE4 carriers has not been intensively investigated in humans so far. Associations with a reduced abundance of Firmicutes as well as a higher abundance of Proteobacteria were reported (31, 70). With regard to the oral microbiota, we found a significantly increased diversity in APOE4 carriers which parallels our findings in patients with AD. In particular, in the at-risk cohort, members of the *Streptococcus* genus were increasingly abundant in contrast to healthy controls. Although these species are part of the physiological oral flora, some strains are associated with caries (71). Furthermore, we found *Streptococcus* to be correlated with low neurocognitive performance in our patients suggesting a role in brain function modulation.

Taken together, two aspects are noteworthy: First, the alterations of the intestinal microbiota that we observed in patients with AD are only partly found in patients with MCI (e.g. higher abundance of Bacteroidetes) and not present in the at-risk group of APOE4 carriers. Second, an increased diversity of the oral microbiota was found in patients with AD and APOE4 carriers.

These aspects suggest that there might be a specific disease stage–dependent interaction between microbial composition and pathological processes in AD. MCI and AD are considered as a continuum of the same pathology, but the “tipping point,” is where the long-lasting pathological processes lead to the clinical manifestation of cognitive decline, and the influencing factors are still not fully understood. The microbiota in the gastrointestinal tract is considered as influencing factors. Our findings indicate that the tipping point might be at the stage of MCI, where the first AD-like alterations in the fecal microbiota including mostly gram-negative species among the phylum Bacteroidetes became apparent. The increased diversity in the oral microbiota might eventually display an early marker for AD as it is detectable in the at-risk and AD cohort, although we could not identify a similar composition of abundant species. Two limitations need to be considered here: The presence of the APOE4 allele as a relative risk factor does not perfectly predict the development of AD (72, 73), thus the definition of APOE4 carriers as an at-risk group for AD does cover only one specific risk factor. In addition, the younger age of the at-risk group has been considered if possible, but may still have influenced the analyses to some extend (74).

Patients with AD further displayed a stronger correlation of the oral and fecal microbial communities, compared with MCI and healthy controls, which may speak for synchronization or spillover between these two compartments. The so-called “oralization” of intestinal communities often stands for a break-down of the oral-gut barrier and has been linked to several inflammatory diseases (75–79). Interestingly, Horvath et al. (80, 81) recently reported that oralization of the gut microbiota is associated with intestinal inflammation and disruption of intestinal barrier function, which may facilitate neuroinflammation and neurodegeneration in AD.

### Functional analysis

In addition to taxonomic differences in response to AD, we also found significant functional differences between healthy and diseased communities on different levels. Disease-associated communities displayed a consistent overrepresentation of functions involved in “Sulfur metabolism” and “Taurine and hypotaurine metabolism.” Competition for sulfur or sulfur metabolites in the gut may reduce uptake and lower sulfur-containing metabolite concentrations which may be involved in AD (82). Taurine and sulfur-containing medication have shown beneficial effects for Alzheimer patients due to their innate antioxidant potential (82). These patterns may be linked to *Solobacterium moorei* which appeared to be a central, yet opportunistic pathogen in the oral microbiota with the capacity to convert cystein to H2S in concert with other oral disease-associated community members like *P. gingivalis* (67, 68).

Interestingly, components of the Curli protein (i.e. csgB, csgE, csgF, and csgG) were more abundant in oral samples of healthy controls compared with patients with AD. The Curli type amyloid protein, which is found in the outer membranes of several *Enterobacteriaceae* is an important factor for biofilm formation (83). It shows similarities to human Aβ and α-synuclein which is implicated in Parkinson's disease and may contribute to pathology through immune activation via systemic or vagal pathways (84). So far, animal experiments showed a higher abundance of Curli in AD and Parkinson's disease mouse models (85, 86); however, substantiation of these patterns in humans is still lacking behind, particularly for AD. Immunological responses to Curli are highly site specific, provoking proinflammatory reactions in systemic infections while leading to intestinal anti-inflammatory and barrier-restoring reactions when administered orally (87, 88). Thus, the lower oral abundance of Curli in AD could represent a differentiating pattern to other neurodegenerative diseases, like Parkinson's disease (89), but requires further validation and mechanistic investigations. We recognize the limited explanatory power of imputed metagenomic functional spectra via PiCRUSt2 in comparison with true shotgun metagenomic sequencing, but emphasize its value as a first insight into the functional characteristics of those communities and the relatively high accuracy of the PICRUSt2 algorithm (90).

### Limitations

Limitations in our study are that only a subset of the participants provided oral samples which was due to the Covid-19 pandemic situation. Longitudinal dietary changes in the course of the disease progression could potentially affect the interpretation of the differences in community compositions between patients and controls (91, 92). Although dietary changes have been associated with patients with AD (93, 94) the impact of diet on microbial composition has not yet been intensively investigated in AD (95). Furthermore, some participants reported the use of antibiotics within the last 6 months. We did not find differences in microbial diversity or abundance of genera between individuals who did or did not use antibiotics in this time span (see Fig. S9). This suggests that the use of antibiotics within the last 6 months did not significantly influence our results. Moreover, although we did not see the effects of different medication on the microbial composition (see Fig. S9), it might still have influenced our results to some extent (96).

### Outlook

Our results provide advanced insights into the host-microbe crosstalk in AD pathology and may contribute to the discovery of diagnostic markers potentially be used as early detection markers and potential therapeutic targets for AD as reported in Ferreiro et al. (97). Determining the mechanistic relationship between the gut and oral microbiota in the progression of AD may lead to novel interventional approaches that alter or restore healthy gut and oral bacterial composition. Validating these findings in longitudinal studies is critical for the translation into clinical benefits.

## Methods

### Ethics

The study was conducted in accordance with the Declaration of Helsinki, and ethical approval was granted by the ethics committee at Kiel University (AZ A103/14, D440/18, and D498/19). All participants provided written informed consent before taking part in the study.

### Cohort characteristics and sampling

#### Patients’ characterization

Patients with AD and MCI were recruited from the outpatient memory clinic and the inpatient clinic at the University Hospital Schleswig-Holstein, Campus Kiel as well as the DIAKO Hospital in Flensburg between October 2018 and April 2021. All patients were diagnosed according to the latest version of diagnostic guidelines (98, 99) and, together with their caregivers, patients were informed about the study in verbal and written form before signing the written informed consent. Healthy elderly controls were recruited via open platforms in the University Hospital Schleswig-Holstein, Campus Kiel as well as from our healthy control database. Participants at-risk were recruited from the University memory clinic as tertiary referral center for risk assessment for developing AD in which course the APOE4 status was tested. Exclusion criteria included any significant neurologic disease except MCI or AD for the patient cohort (including active major psychiatric disease such as alcohol/substance dependence, major depression, or other neurodegenerative disease) or any significant acute illness (including major gastrointestinal tract surgery in the past 2 years, active uncontrolled gastrointestinal disorder or active inflammatory bowel disease, persistent infectious gastroenteritis, or chronic diarrhea). Microbiome-specific exclusion criteria were the current usage of antibiotics, changes in medical treatment within the previous 3 months (including no use of corticosteroid or immunosuppressive agents), or major dietary changes during previous months. All participants completed a questionnaire addressing lifestyle and nutritional behavior as well as medication at the time of stool and saliva sampling.

For neurocognitive assessment, we used the MMSE, MoCA, and MRDS. The MoCA test was designed as a rapid screening instrument for mild cognitive dysfunction and assesses different cognitive domains such as attention and concentration, executive functions, memory, language, visuoconstructive skills, conceptual thinking, calculations, and orientation (range: 0–30, <26 MCI/AD). The MMSE is a widely used quantitative screening assessment for cognitive impairment with a score under 24 indicating cognitive impairment associated with dementia in 79% of cases (100, 101). MRDS is an additional assessment used for detection of cognitive impairment in the elderly population supplementing the other metrics by examining different cognitive domains like attention, perseveration/initiation, construction, combination, and memory (102, 103). We defined *z*-scores of the total MRDS score using the most current normative data available (104). In order to harmonize data from different cognitive assessments, we used the individual *z*-scores of the 3 tests (called cognition) for further analysis. Embedded in the clinical diagnostic routine, CFS biomarker as Aβ_1–42_, tau-, and p-tau were evaluated. In order to incorporate both pathophysiological aspects of AD in 1 variable, we used the ratio of p-tau and Aβ_1–42_ (p-tau/Aβ_1–42_) for correlative analysis. Blood was taken within the clinical routine to assess general inflammatory parameters including CRP. Furthermore, we performed cerebral MRI on patients with AD and MCI and evaluated the MTA score, a score to visually graduate the MTA in patients with AD and MCI by means of loss of hippocampal height, width of the choroidal fissure, and width of the temporal horn (105). The score ranges from 0 (no mediotemporal atrophy) to 4 (severe loss of mediotemporal volume) and was validated for diagnosis of AD in a research context (106). The visual inspection was performed by three clinical evaluators (board-certified neurologist and two radiologists, T.B., L.P.S., and S.A.) experienced in detecting mediotemporal atrophy and scoring the MTA score blinded to the patient's diagnosis.

#### Sampling of fecal and oral microbiome

All participants resided at home and provided fecal samples collected at home by means of a prepared collection kit including two stool tubes (Sarstedt sterile stool tube, 76 × 20 mm), 1 with 5 mL RNAlater RNA stabilization solution (Invitrogen), the other without puffer solution. For further analysis, only samples without stabilization solution were used. Samples were sent back within 24 h. Fecal samples were immediately frozen at −80°C after arrival at the department of Neurology. Swab samples (Sarstedt forensic swab tube with ventilation membrane, 93 mm, ISO 18385) were taken by a physician or a trained assistant at the outpatient visit of the participant by swabbing the left and right inner cheek for 30 s and directly frozen at −80°C on average of 1 week before fecal sampling. At the time of fecal sample collection, participants completed a questionnaire containing information about dietary habits, medication, recent antibiotic, or probiotic use, as well as current and past gastrointestinal and metabolic conditions.

#### Stool and swab sample processing and sequencing

DNA of stool samples were extracted using the QIAamp DNA fast stool mini kit automated on the QIAcube (Qiagen, Hilden, Germany). Material was transferred to 0.70 mm Garnet Bead tubes (Qiagen) filled with 1.1 mL InhibitEx lysis buffer. For swab samples, QIAamp UCP Pathogen mini kit automated on the QIAcube was used. The swab was therefore transferred to a Pathogen Lysis Tube S filled with 0.65 mL ATL buffer (incl. DX) and incubated for 10 min at 56°C with continuous shaking at 600 rpm. Bead beating for both sample types was performed using a SpeedMill PLUS (Analytik Jena, Jena, Germany) for 45 s at 50 Hz with subsequent continuation of the manufacturer's protocol. Extracted DNA was stored at −20°C before PCR amplification. Blank extraction controls were included during extraction of samples, PCR, and sequencing. Amplicon sequencing of variable regions 1 and 2 of the 16S rRNA gene was done as described in detail earlier (107).

### Sequence data processing

Data processing was performed using DADA2 v1.10 (108), via the workflow for big datasets (https://benjjneb.github.io/dada2/bigdata.html). This resulted in abundance tables of ASVs according to a workflow adjusted for V1-V2 region, which can be found here: https://github.com/mruehlemann/ikmb_amplicon_processing/blob/master/dada2_16S_workow_with_AR.R. Resulting ASVs underwent taxonomic annotation using the Bayesian classifier provided in DADA2 and using the Ribosomal Database Project version 16 release (109). One sample with <10,000 sequences was not considered for further analysis. The representative ASV sequences were aligned using the Nearest Alignment Space Termination (NAST) algorithm and filtered for informative sites (excluding constant gaps/bases) in mothur (110, 111). Phylogenetic trees were constructed via FastTree v2.1 (112) using the “CAT” substitution model with Γ-correction and improved accuracy (longer initial tree search [-spr 4], slower initial tree search [-slownni], more exhaustive tree search [-mlacc 2]).

ASV sequences and abundance matrices were transformed into biom format (R package “biomformat” v1.24.0) (113) and processed via the standard pipeline of PICRUSt2 (v2.5.0) (90). Functional information was imputed in the form of KEGG functional orthologs (114) and used for further analyses. Sequences which poorly aligned or were too distant from the least common ancestor (Nearest Sequenced Taxon Index [NSTI] value) were removed (16 ASVs).

### Statistical analyses of microbiota analyses

The R package “vegan” (v2.5-1) (115) was used to investigate alpha diversity in this study. Alpha diversity of the samples was measured by Chao1 and Shannon diversity. Phylogenetic alpha diversity was measured by unweighted NRI and NTI as implemented in “picante” (v1.8.2) (35, 116). The association between microbial diversity and compared groups (controls vs. AD, MCI, or at-risk participants) were tested via linear regression models. Linear regression models were run in *R* (117) via the lm function from “stats” package. Age and sex were included as covariates in all statistical tests and normality was checked via the Shapiro–Wilk test.

Trend analyses of taxa monotonically correlating with disease severity were performed on healthy individuals (including at-risk individuals), MCI, and AD patients. The health condition was recorded as a severity gradient (controls and at-risk = 0, MCI = 1, AD = 2). Taxon abundances were *clr* transformed (R package compositions) and correlated with the severity score by linear models, incorporating age and sex as covariates. Results were visualized via heatmaps which were generated using the R package “complexHeatmap” v2.14.0 (including Ward clustering).

For analysis on beta-diversity, NMDS was performed with Bray–Curtis dissimilarity as implemented via *metaMDS* in the R package “vegan.” Bray–Curtis distance matrices based on the microbial communities in all samples were generated using the R package “phyloseq” v1.22.3 (118). A permutational multivariate analysis of variance (PERMANOVA) (119) was then performed on the distance matrix to assess the effects of status (healthy controls vs AD, MCI, or at-risk participants) on variance between microbial communities. The PERMANOVA was performed based on the *adonis* function of the R package “vegan,” with 999 permutations. *Betadisper* function was used to test for homogeneity of variances between groups.

Differential abundance of taxa between AD, MCI, and control groups was determined at the ASV level using the “DESeq2” package in R (120), for each ASV which was covered by at least 50 read pairs and showed a minimum prevalence of 5% in all samples. Missing data are included as partial data in “DESeq2” models. Age and sex were included as covariates. Results were expressed as log_2_ fold change in AD and MCI participants relative to control participants. False discovery rate (FDR) was used for multiple testing correction. Similar techniques were applied for the analyses of differentially abundant functions, including subject age and sex as covariates, and performing negative binomial models with Wald tests as implemented in “DESeq2” (including automated effect filtering) to derive significance (FDR corrected). Enrichment analyses were performed on significant K-numbers for the respective pairwise comparisons and associations via “clusterProfiler” (v4.4.4) using the *enrichKEGG* function (121, 122) (KEGG database access: 2022 December 15, *p*-values are FDR corrected). Heatmaps were generated using the R package “complexHeatmap” v2.14.0 (123).

For correlation analysis, we used linear regression models. Linear regression models were run in R (117) via the lm function from “stats” package, after *clr* transformation from compositions package, ASV abundances were defined as outcome variable and AD score levels, sex, and age as the dependent variables (FDR corrected). The correlation matrix was plotted as a heatmap including hierarchical clustering of samples and variables via average linkage clustering (based on the Euclidean distance measurements) (123).

Weighted coabundance networks were generated based on the correlation of ASV abundances (present >5% of samples). Networks were generated individually for the fecal and oral samples, via graphical models (Meinshausen–Buhlmann's neighborhood selection) as implemented in “SpiecEasi” v1.1.2 (124)⁠. The resulting networks were of sizes 1,094 and 830 nodes after filtering for edges present in ≥80% of the network subsamples (bounded StARS selection, 50 permutations, and 50 lambda paths) (125, 126)⁠. Node-based importance measures (degree, betweenness, and PageRank index) were calculated in “igraph” v1.2.4.1 (127–130). To assess whether bacteria were more important than expected by chance, observed centralities were compared against a permuted set of networks (1,000 times) via one-sided *Z*-tests (FDR corrected). Indicator species analysis as implemented via the function *multipatt* in the “indicspecies” package (v1.7.12) was used to detect predictive taxon for the respective patient characteristics (36)⁠.

## Supplementary Material

pgad427_Supplementary_Data

## Data Availability

The datasets generated and analyzed during the current study are available at the European Nucleotide Archive (ENA, Project accession number: PRJEB59009), please see https://www.ebi.ac.uk/ena/browser/search. Codes are available at: https://github.com/trocialba/Alzheimer-Project.

## Preprints

This manuscript was posted on a preprint: https://doi:10.1101/2023.03.27.23287778.
