# Supplementary material for: Disease- and stage-specific alterations of the oral and fecal microbiota in Alzheimer's disease
Source: PNAS Nexus. 2023 Dec 11;3(1):pgad427. doi: 10.1093/pnasnexus/pgad427 (PMC10776369; doi:10.1093/pnasnexus/pgad427)
Supplement: pgad427_Supplementary_Data [file pgad427_supplementary_data.zip › PNASNEXUS-PNASNEXUS-2023-00320RR-s01.pdf]

Supporting Information for

Disease and stage specific alterations of the oral and fecal microbiota in Alzheimer's disease

Alba Troci[1]*, Sarah Philippen[2]*, Philipp Rausch[1]*, Julius Rave[2], Gina Weyland[2], Katharina Niemann[2], Katharina Jessen[2], Lars-Patrick Schmill[3], Schekeb Aludin[3], Andre Franke[1], Daniela Berg[2], Corinna Bang[1]*, Thorsten Bartsch[2]*#

[1] Institute of Clinical Molecular Biology, Kiel University, Kiel, Germany
[2] Department of Neurology, Memory Disorders and Plasticity Group, University Hospital Schleswig-Holstein, Campus Kiel, Arnold-Heller-Strasse 3, 24105, Kiel, Germany
[3] Department of Radiology and Neuroradiology, University Hospital Schleswig-Holstein, Kiel, Arnold-Heller-Strasse 3, 24105, Kiel, Germany

*equal contribution

[#]corresonding author: Thorsten Bartsch
Email: t.bartsch@neurologie.uni-kiel.de

This PDF file includes:

Figures S1 to S9
Tables S1 to S11

Supplemental Figures

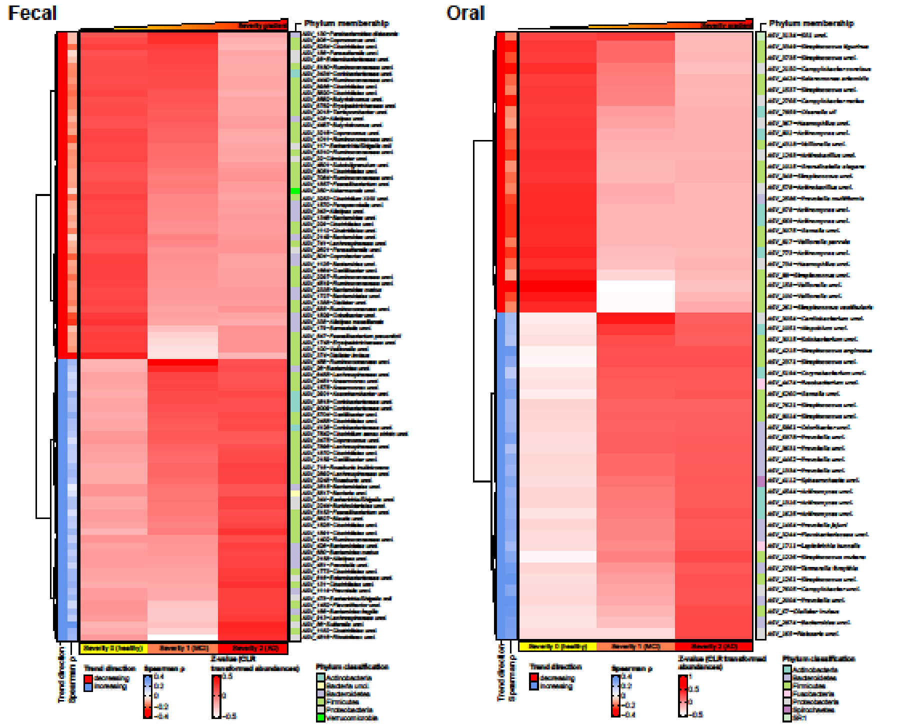

Fig. S1. Heatmaps visualize the average CLR transformed abundances (groupwise Zz -valuetranformed) of nominally significant ASVs (P≤0.05) associated with disease severity (controls & at-risk=0, MCI=1, AD=2; top and bottom color bars) in the fecal (A) and oral (B) microbial community. Left color bars highlight the trend direction (increasing/decreasing with severity) and naive Spearman correlation coefficient (ρ). Right color bars show the taxonomic membership of associated ASVs at the phylum level and ASVs are arranged following Ward clustering based on euclidean distances (fecal: N(0)=63, N(1)=15, N(2)=63; oral: N(0)=25, N(1)=7, N(2)=43).Abbreviations: MCI, mild cognitive impairment; AD, Alzheimer’s disease

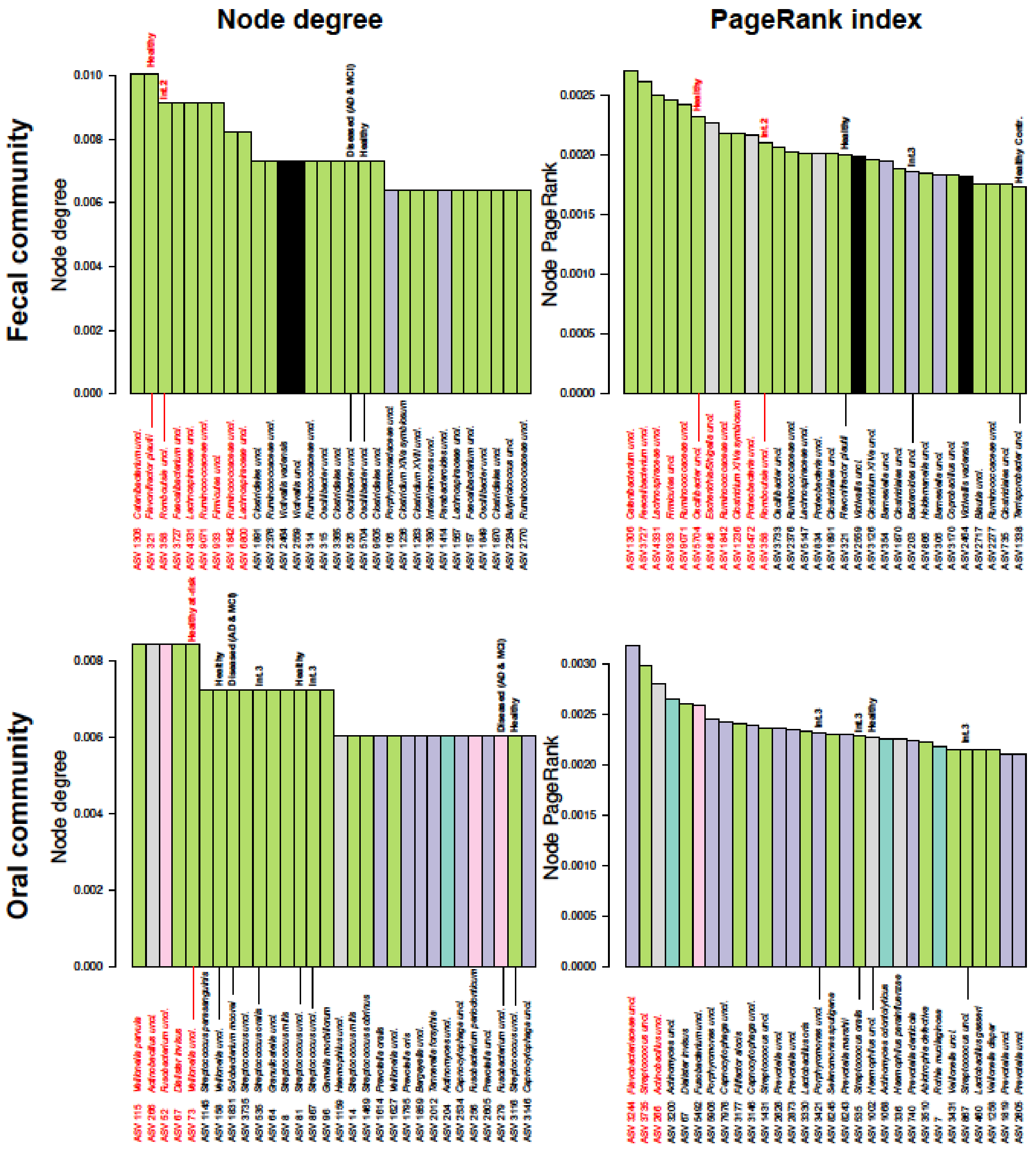

Fig. S2. Centrality measures of the 30 most important nodes of the respective importance measures based on the relative number of node connections/correlations to other taxa (node degree) or their general importance based on linkage to other important taxa (PageRank index). Red highlights network members with a higher importance than expected by chance, based on a Z-test against 10’000 randomized networks (PFDR ≤ 0.05). Additional mark ups indicate the taxon associations as detected by indicator species analyses (PFDR ≤ 0.05, see Table S5). Abbreviations: H., controls & at-risk individuals; Dis., MCI & AD; Int. 2, intensity 2, Int.3, intensity 3.

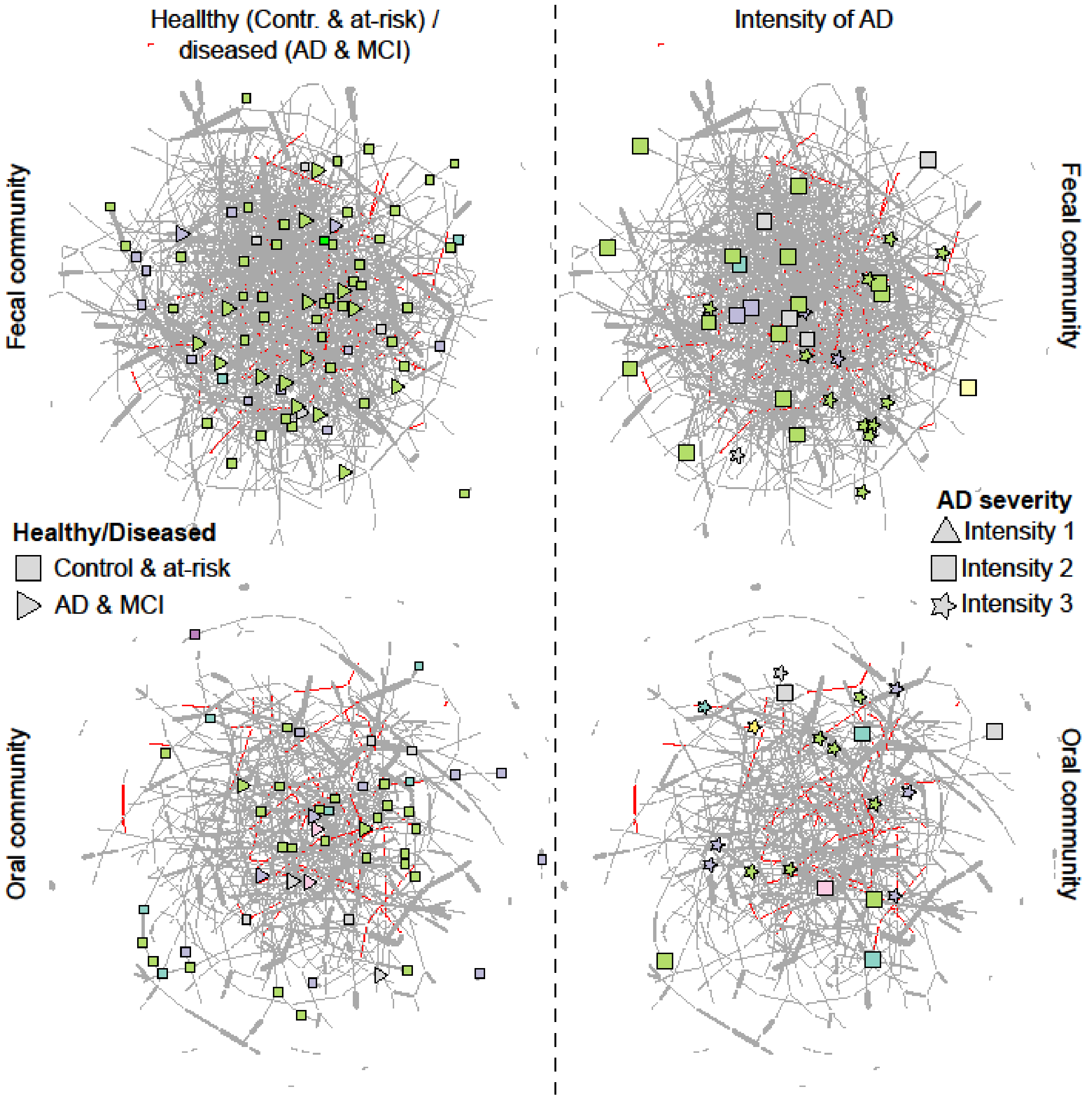

Fig. S3. Fecal and oral correlation networks based on the SpiecEasi algorithm. Only nodes matching significant indicator species in the respective dataset are shown and highlighted by factor level specific symbols (Healthy (Contr. & at-risk)/Diseased (MCI & AD): ■-healthy, ►-diseased; AD clinical intensity▲-intensity 1, ■-intensity 2, □★-intensity 3). Abbreviations: MCI, mild cognitive impairment; AD, Alzheimer's disease; Contr., Controls.

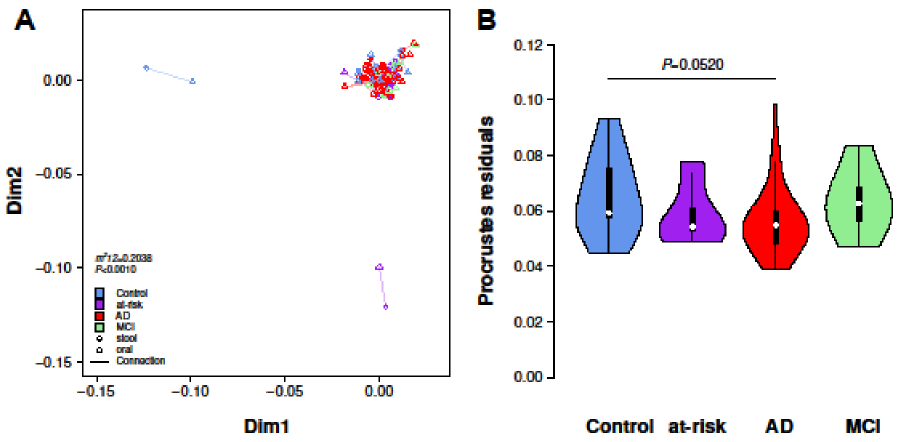

Fig. S4. (A) Procrustes analysis performed using the Bray-Curtis distances derived from oral and fecal ASV abundances. Significance was derived from 10,000 permutations. (B) Residuals were derived from the fit of the two configurations after rotation and scaling to maximum similarity. Larger residuals imply larger differences in the patterns of the oral and fecal microbial communities, which were assessed via pairwise Wilcoxon-tests. Abbreviations: AD, Alzheimer’s disease; MCI, mild cognitive impairment.

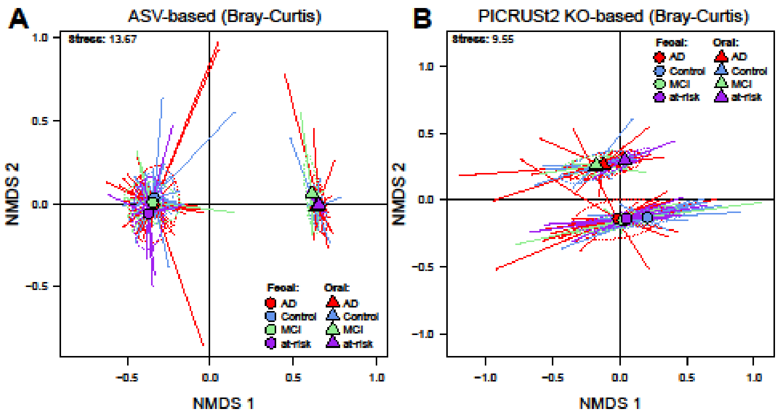

Fig. S5. (A) Non-metric multidimensional scaling (NMDS) visualizing the differentiation of bacterial communities based on the differential ASV abundances (Bray-Curtis distance) between samples of healthy controls, healthy at-risk individuals, and patients with AD or MCI in fecal and oral samples (fecal vs. oral: $F_{1,214}=23.685$, $P<0.00001$, $R2=0.09965$, PERMANOVA). (B) NMDS displaying functional differences between healthy and diseased individuals based on the Bray-Curtis distance calculated from KO abundances in fecal and oral samples (fecal vs. oral: $F_{1,214}=67.557$, $P<0.00001$, $R2=0.23994$, PERMANOVA). Abbreviations: MCI, mild cognitive impairment; AD, Alzheimer’s disease.

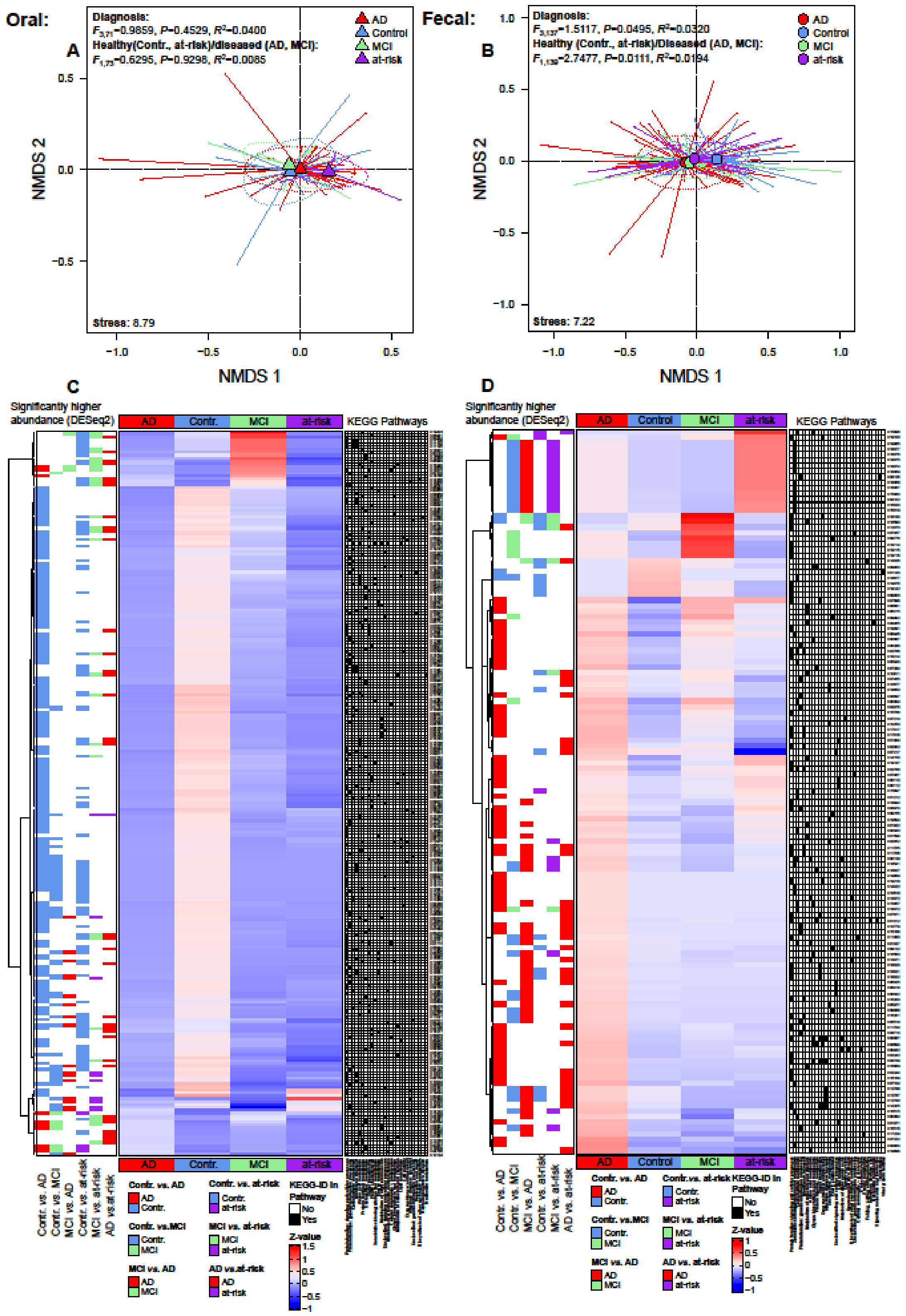

Fig. S6. Community differences based on functional predictions (Bray-Curtis) derived from PICRUSt2 in oral and fecal microbial communities. Significance of differences with respect to the different diagnoses and health conditions were ascertained via PERMANOVA. Heatmaps display significantly differentially abundant functional predictions (DESeq2, Wald-test, PFDR ≤ 0.01). Displayed are average Z-values of VST transformed KO abundances for the respective health conditions. Left color/ annotation bar highlights the association of the respective function in the different comparisons (direction of fold abundance change in Controls vs AD, Controls vs MCI, MCI vs. AD), which are arranged following Ward clustering based on euclidean distances. Right annotation matrix displays the membership of the

function in the respective pathways based on KEGG categories. Abbreviations: AD, Alzheimer's disease; MCI, mild cognitive impairment; Contr., Controls.

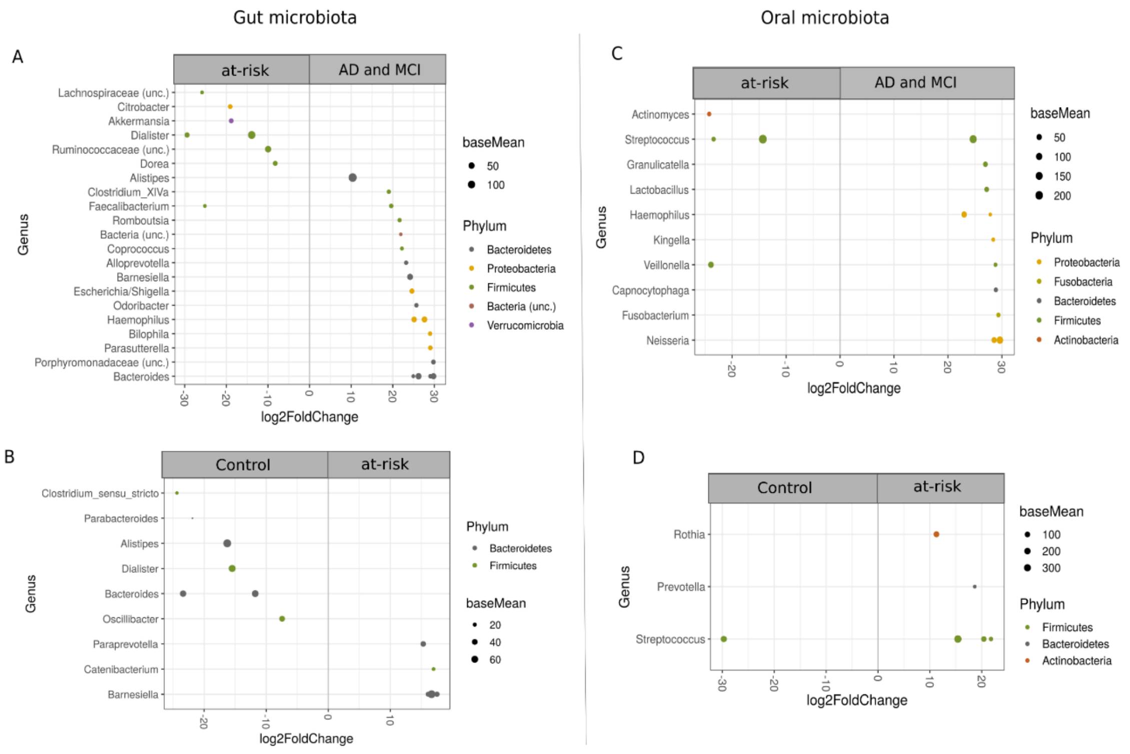

Fig. S7. Significant abundant taxa between AD and MCI patients and at-risk group in stool (A) and swab samples (C) were calculated by using DESeq2 (at least 50 read pairs/sample, prevalence ≥ 5%). The right part of the plots shows taxa, which were more abundant in AD and MCI patients and in the left part those with higher abundance in the at-risk group. Significant abundant taxa between at-risk group and healthy controls in stool (B) and oral swab samples (D) were calculated by using DESeq2 (at least 50 read pairs/sample, prevalence ≥ ≥ 5%). The right part of the plots shows taxa, which were more abundant in the at-risk group and in the left part those with higher abundance in healthy controls. Abbreviations: AD, Alzheimer's disease; MCI, mild cognitive impairment.

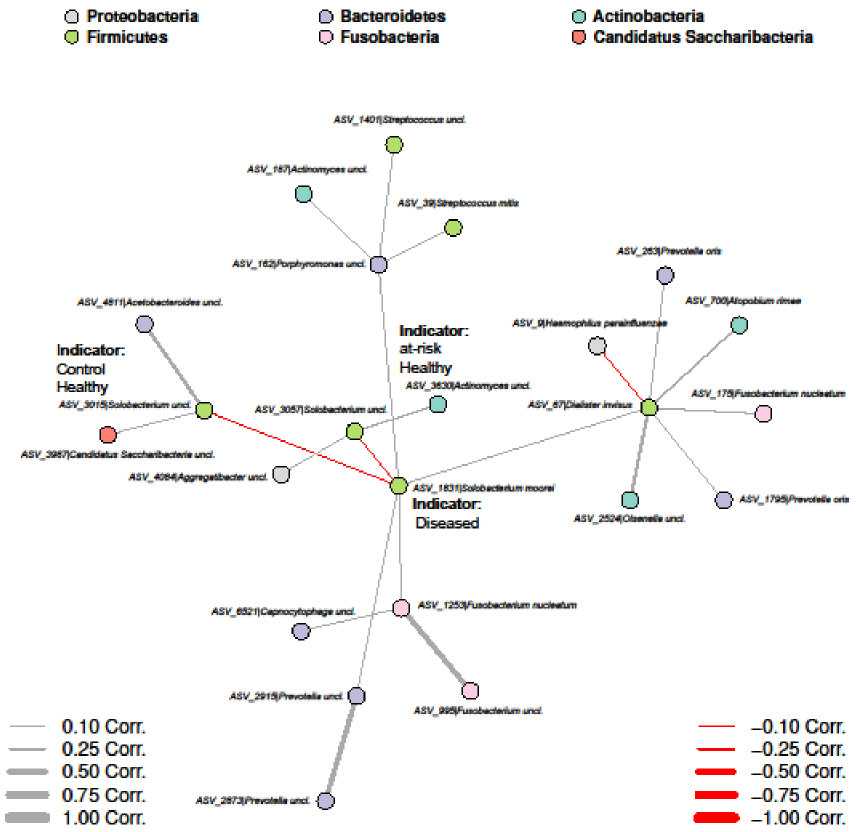

Fig. S8. Subnetwork of the first and second order neighborhood of Solobacterium moorei (ASV_1831) in the oral community network, including direct positive associations between S. moorei and ASVs belonging to e.g.Porphyromonas and Fusobacterium. Indicator status of the respective taxaon in is indicated in the subnetwork are indicated. Abbreviations: Corr., SPIEC-EASI correlation/inverse covariance.

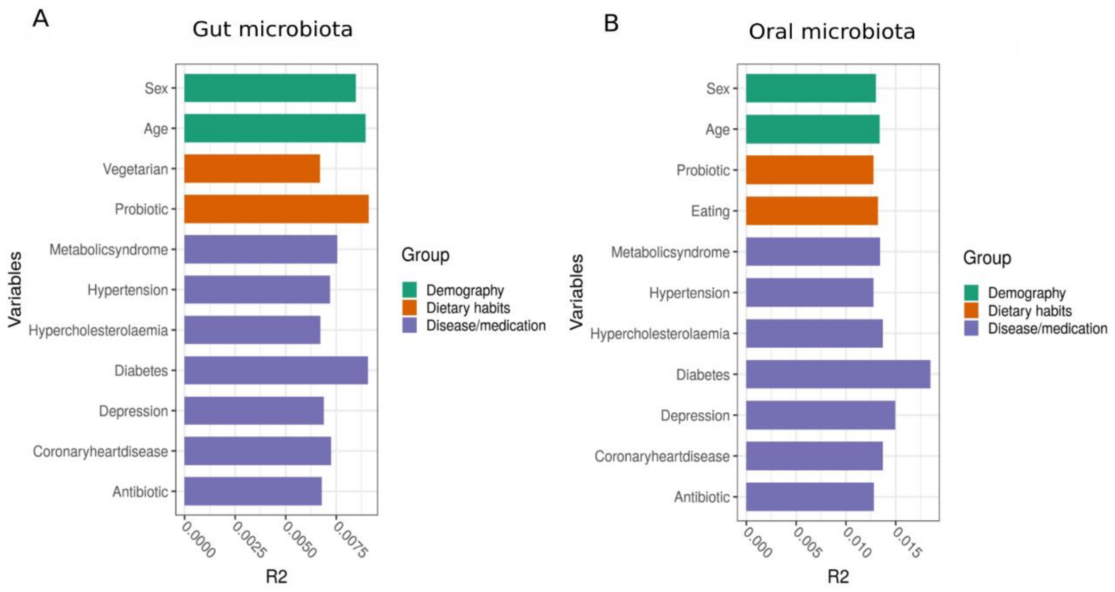

Fig. S9. Significance and explained variance of 11 microbiome covariates modeled by adonis across all data types. Horizontal bars show the amount of variance (R2) explained by each covariate in the model as determined by adonis. Covariates are colored based on the overall metadata group. Adonis test showed no significant covariates (PFDR > 0.05). (A) Microbiota profiles at the ASV level of the gut microbiota. (B) Microbiota profiles at the ASV level of the oral microbiota.

Table S1. Adonis test results on fecal and oral microbiota (ASV based Bray-Curtis distances).

Table S2. Differential abundance test results with DESeq2 (AD vs. HC, AD vs. MCI).

Table S3. Results of ASV based trend analyses (CLR transformed) focusing on disease severity (healthy controls & at-risk=0, MCI=1, AD=2).

Table S4. Results of ASV based indicator species analyses.

Table S5. Significant network importances/centralities in the oral and fecal correlation networks (permutation test based).

Table S6. Correlation test results between ASV abundance and biomarkers.

Table S7. Adonis test results on fecal and oral microbiota (KO based Bray-Curtis distances).

Table S8. Results of differential abundance tests (DESeq2) based on PICRUSt2 KOs in the fecal microbiota (Contr. vs. MCI vs. AD vs. at-risk; PFDR ≤ 0.01).

Table S9. Results of differential abundance tests (DESeq2) based on PICRUSt2 KOs in the oral microbiota (Contr. vs. MCI vs. AD vs. at-risk; PFDR ≤ 0.01).

Table S10. Results of differential abundance tests (DESeq2) based on PICRUSt2 KOs in the fecal and oral microbiota (Healthy (Contr. & at-risk) vs. Diseased (MCI & AD); PFDR ≤ 0.01).

Table S11. Enrichment of differentially abundant PICRUSt2 KOs with respect to diagnosis (Contr. vs. MCI vs. AD vs. at-risk) and health status (Healthy (Contr. & at-risk) vs. Diseased (MCI & AD)).
