# Supplementary material for: Disease- and stage-specific alterations of the oral and fecal microbiota in Alzheimer's disease
Source: PNAS Nexus. 2023 Dec 11;3(1):pgad427. doi: 10.1093/pnasnexus/pgad427 (PMC10776369; doi:10.1093/pnasnexus/pgad427)
Supplement: pgad427_Supplementary_Data [file pgad427_supplementary_data.zip › PNASNEXUS-PNASNEXUS-2023-00320RR-s04.pdf]

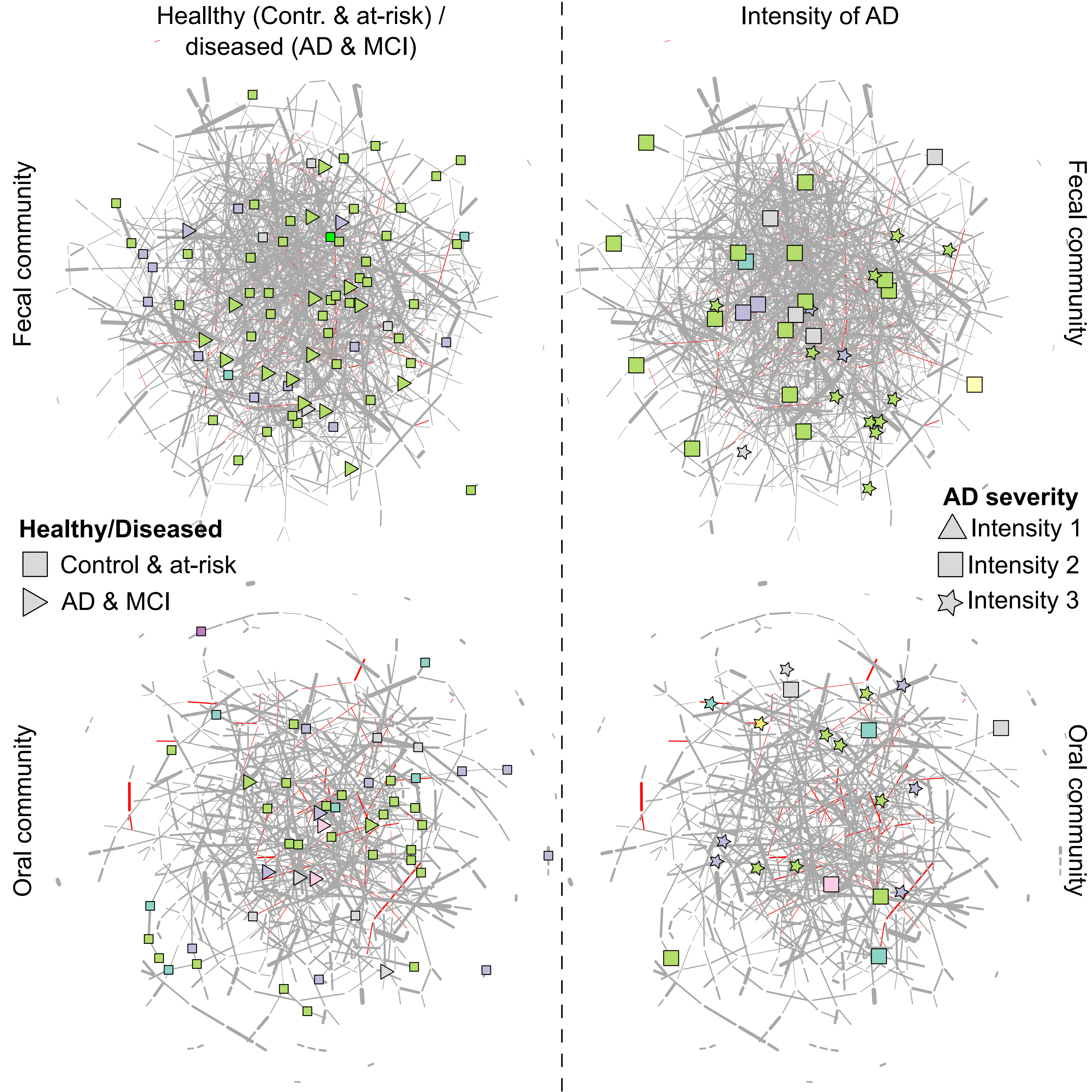
Heallthy (Contr. & at-risk) / diseased (AD & MCI)
Intensity of AD
Fecal community
Fecal community
Oral community
Oral community
Healthy/Diseased
Control & at-risk
AD & MCI
AD severity
Intensity 1
Intensity 2
Intensity 3
