# Supplementary material for: Disease- and stage-specific alterations of the oral and fecal microbiota in Alzheimer's disease
Source: PNAS Nexus. 2023 Dec 11;3(1):pgad427. doi: 10.1093/pnasnexus/pgad427 (PMC10776369; doi:10.1093/pnasnexus/pgad427)
Supplement: pgad427_Supplementary_Data [file pgad427_supplementary_data.zip › PNASNEXUS-PNASNEXUS-2023-00320RR-s09.pdf]

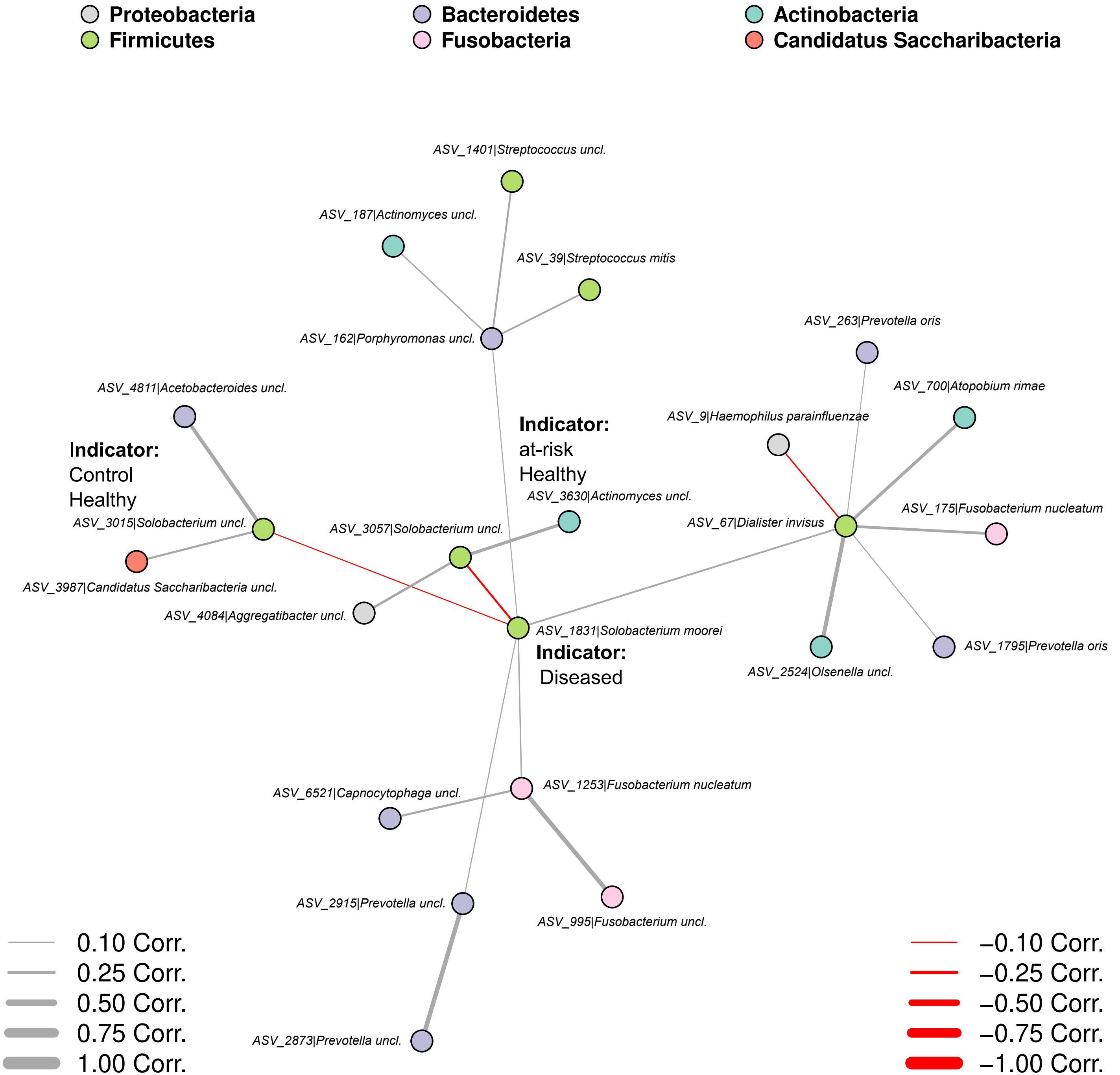
Proteobacteria
Firmicutes
Bacteroidetes
Fusobacteria
Actinobacteria
Candidatus Saccharibacteria
ASV_1401|Streptococcus uncl.
ASV_187|Actinomyces uncl.
ASV_39|Streptococcus mitis
ASV_162|Porphyromonas uncl.
ASV_263|Prevotella oris
ASV_700|Atopobium rimae
ASV_4811|Acetobacteroides uncl.
Indicator:
at-risk
Healthy
ASV_9|Haemophilus parainfluenzae
Indicator:
Control
Healthy
ASV_3630|Actinomyces uncl.
ASV_175|Fusobacterium nucleatum
ASV_3015|Solobacterium uncl.
ASV_3057|Solobacterium uncl.
ASV_67|Dialister invisus
ASV_3987|Candidatus Saccharibacteria uncl.
ASV_4084|Aggregatibacter uncl.
ASV_1831|Solobacterium moorei
ASV_1795|Prevotella oris
Indicator:
Diseased
ASV_2524|Olsenella uncl.
ASV_1253|Fusobacterium nucleatum
ASV_6521|Capnocytophaga uncl.
ASV_2915|Prevotella uncl.
ASV_995|Fusobacterium uncl.
ASV_2873|Prevotella uncl.
0.10 Corr.
0.25 Corr.
0.50 Corr.
0.75 Corr.
1.00 Corr.
−0.10 Corr.
−0.25 Corr.
−0.50 Corr.
−0.75 Corr.
−1.00 Corr.
