# Supplementary figures and images for: Disease- and stage-specific alterations of the oral and fecal microbiota in Alzheimer's disease
Source: PNAS Nexus. 2023 Dec 11;3(1):pgad427. doi: 10.1093/pnasnexus/pgad427 (PMC10776369; doi:10.1093/pnasnexus/pgad427)

# Fecal

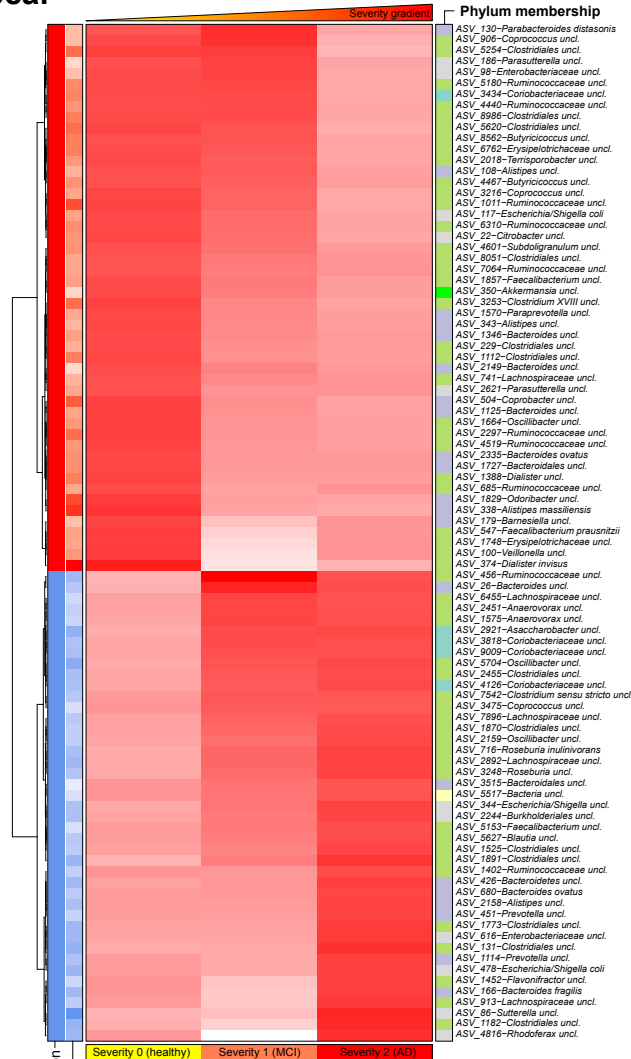

# Oral

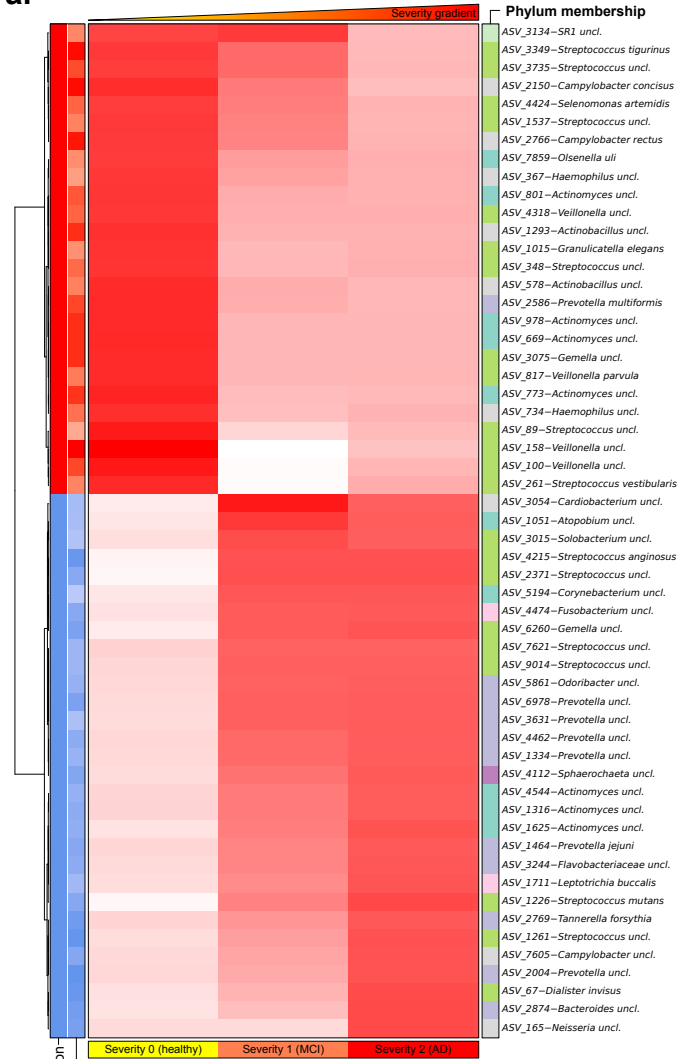

Supplement: pgad427_Supplementary_Data [file pgad427_supplementary_data.zip › PNASNEXUS-PNASNEXUS-2023-00320RR-s02.pdf]

# Oral community

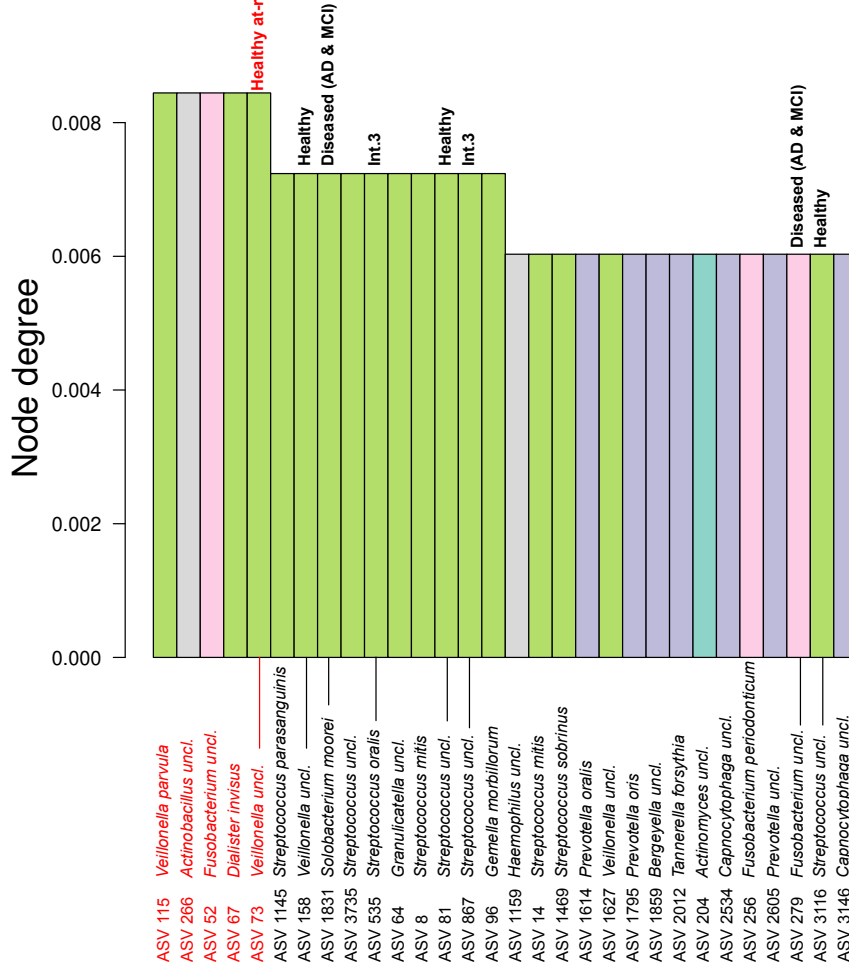

# Fecal community

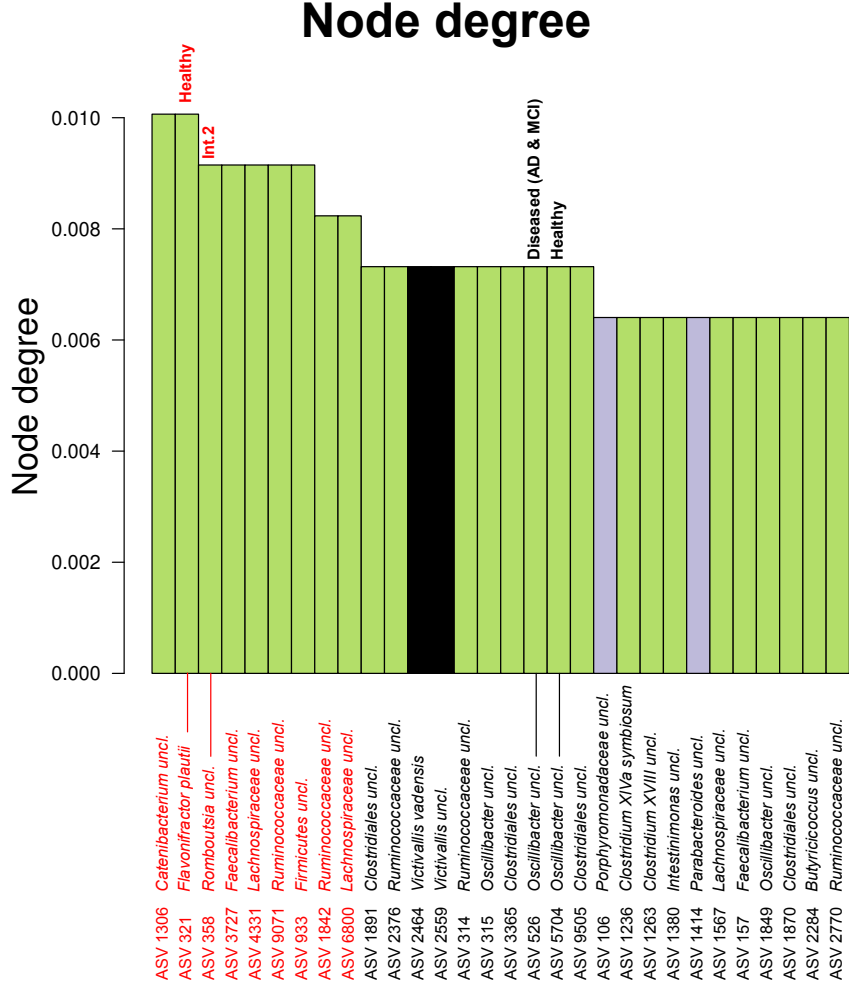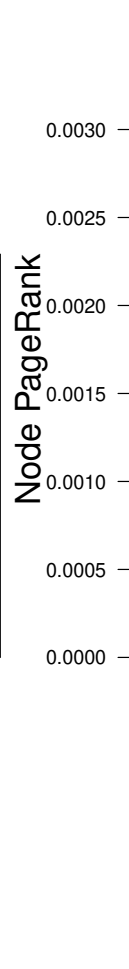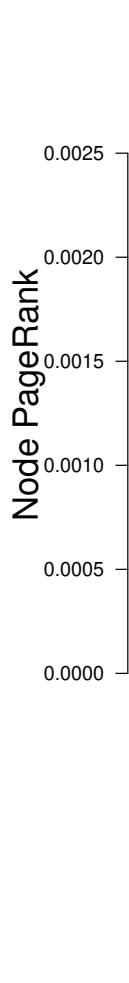

Supplement: pgad427_Supplementary_Data [file pgad427_supplementary_data.zip › PNASNEXUS-PNASNEXUS-2023-00320RR-s03.pdf]

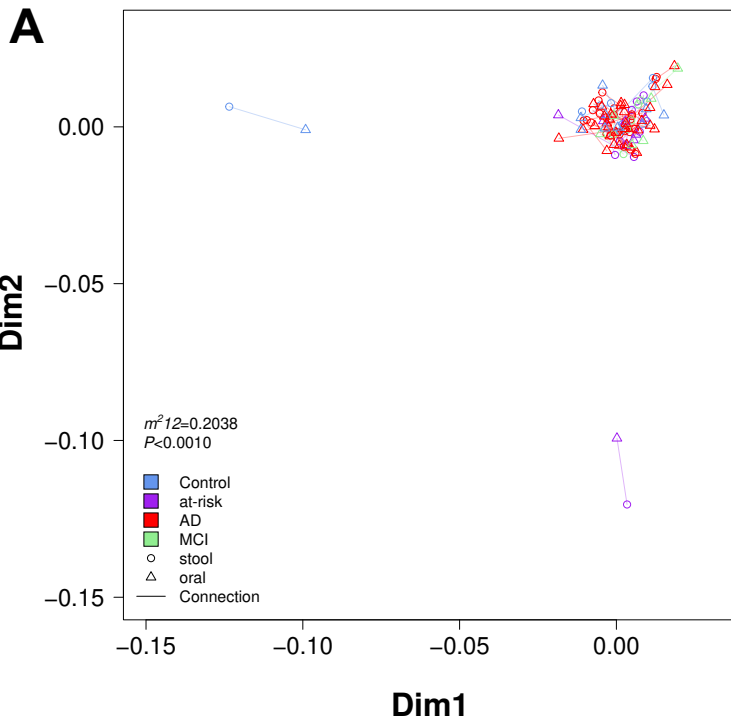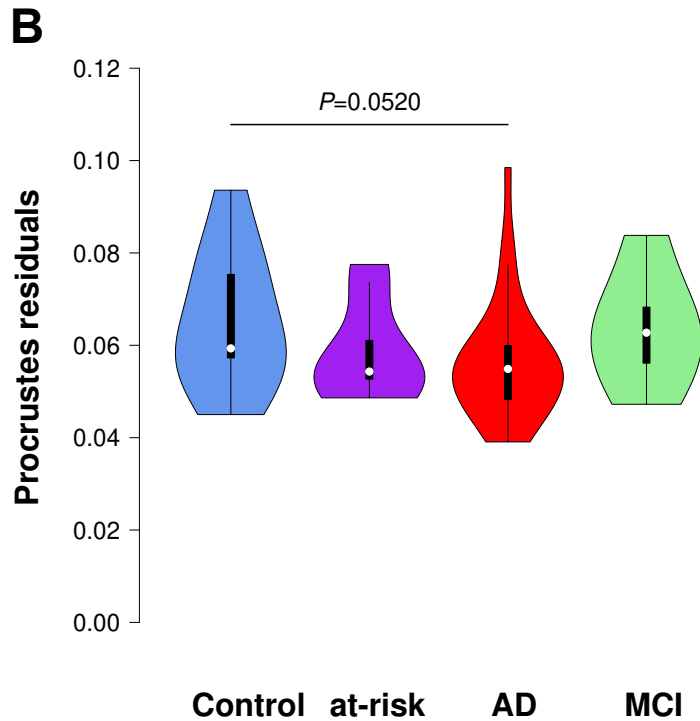

Supplement: pgad427_Supplementary_Data [file pgad427_supplementary_data.zip › PNASNEXUS-PNASNEXUS-2023-00320RR-s05.pdf]

**A****ASV-based (Bray-Curtis)**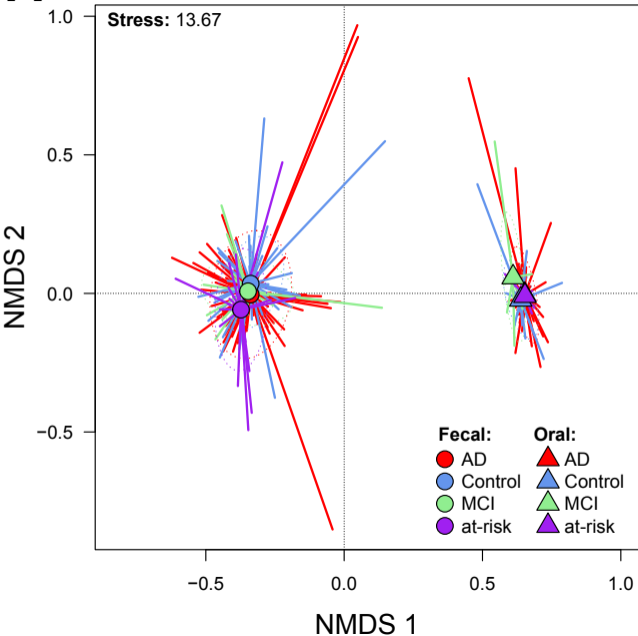**B****PICRUSt2 KO-based (Bray-Curtis)**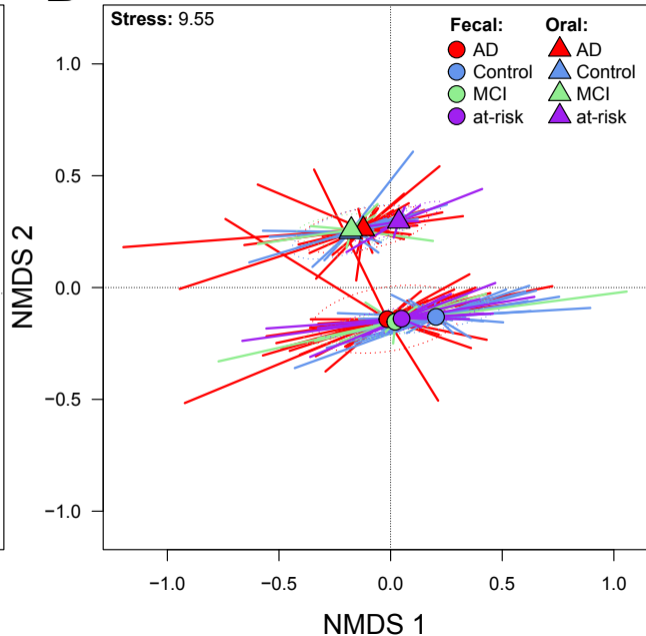

Supplement: pgad427_Supplementary_Data [file pgad427_supplementary_data.zip › PNASNEXUS-PNASNEXUS-2023-00320RR-s06.pdf]

Oral:

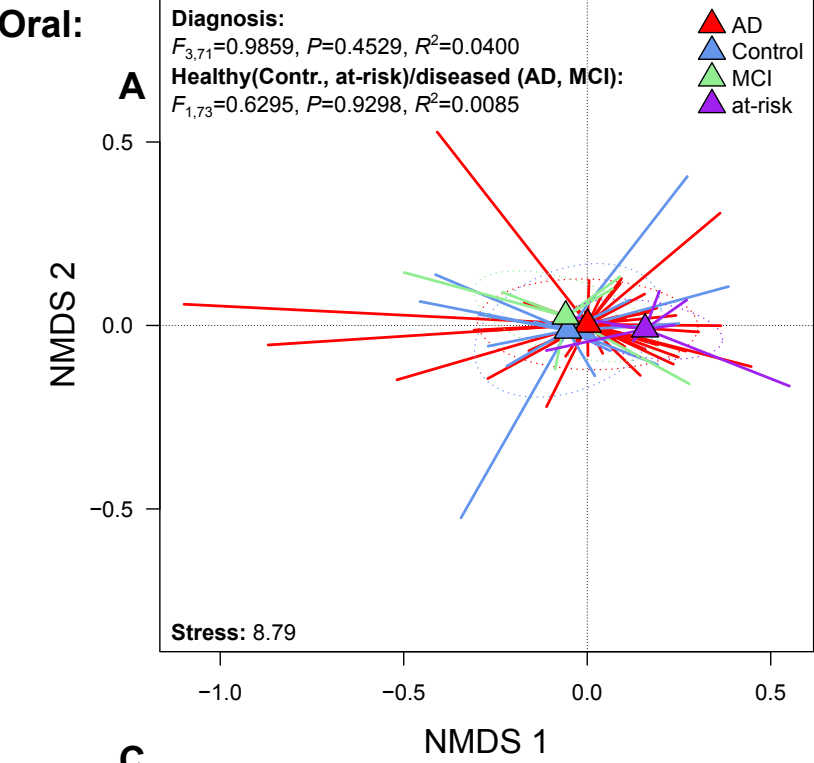

Fecal:

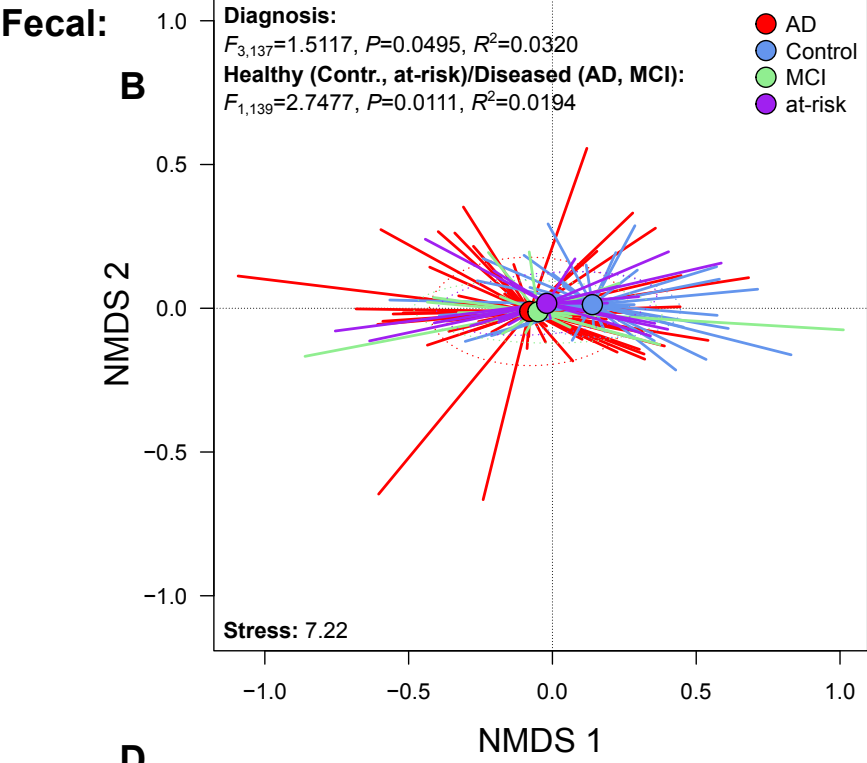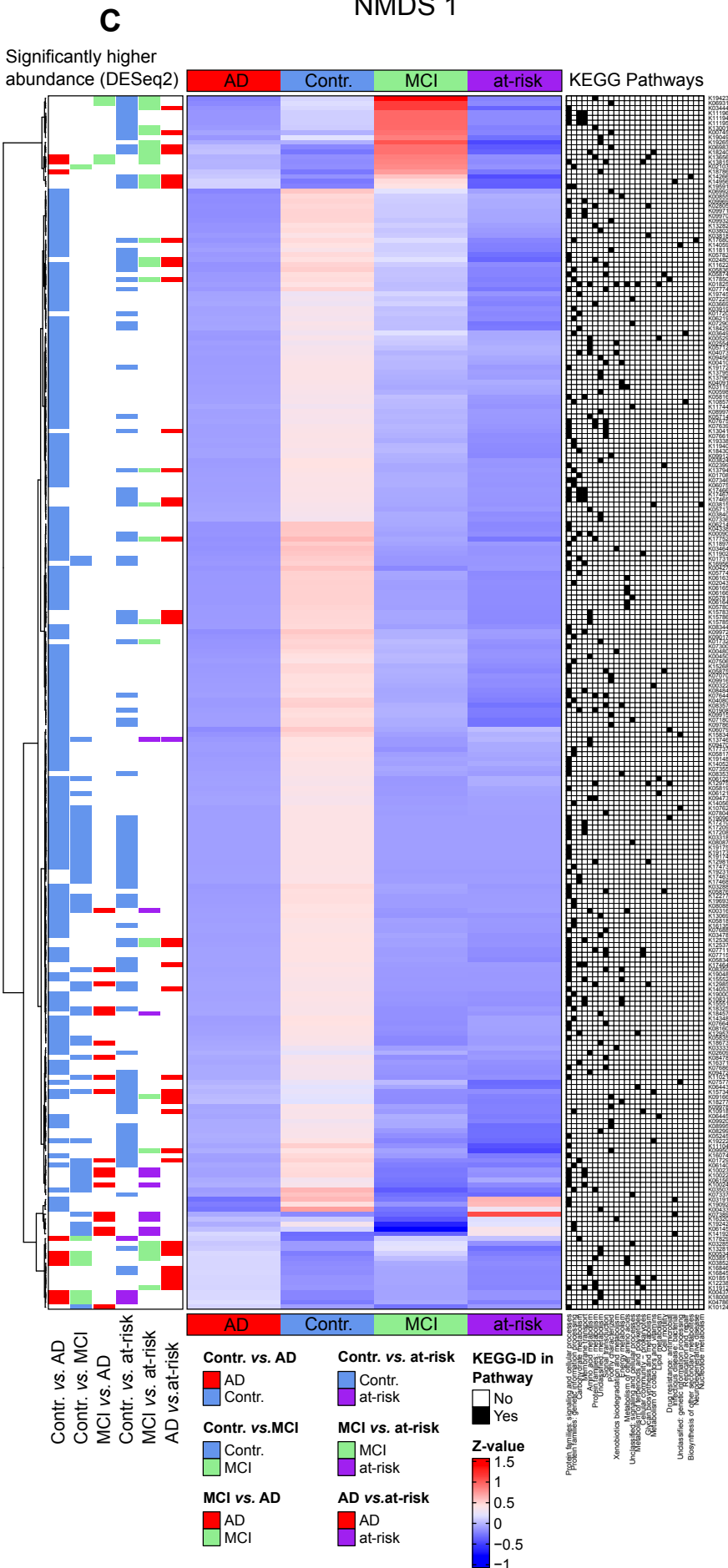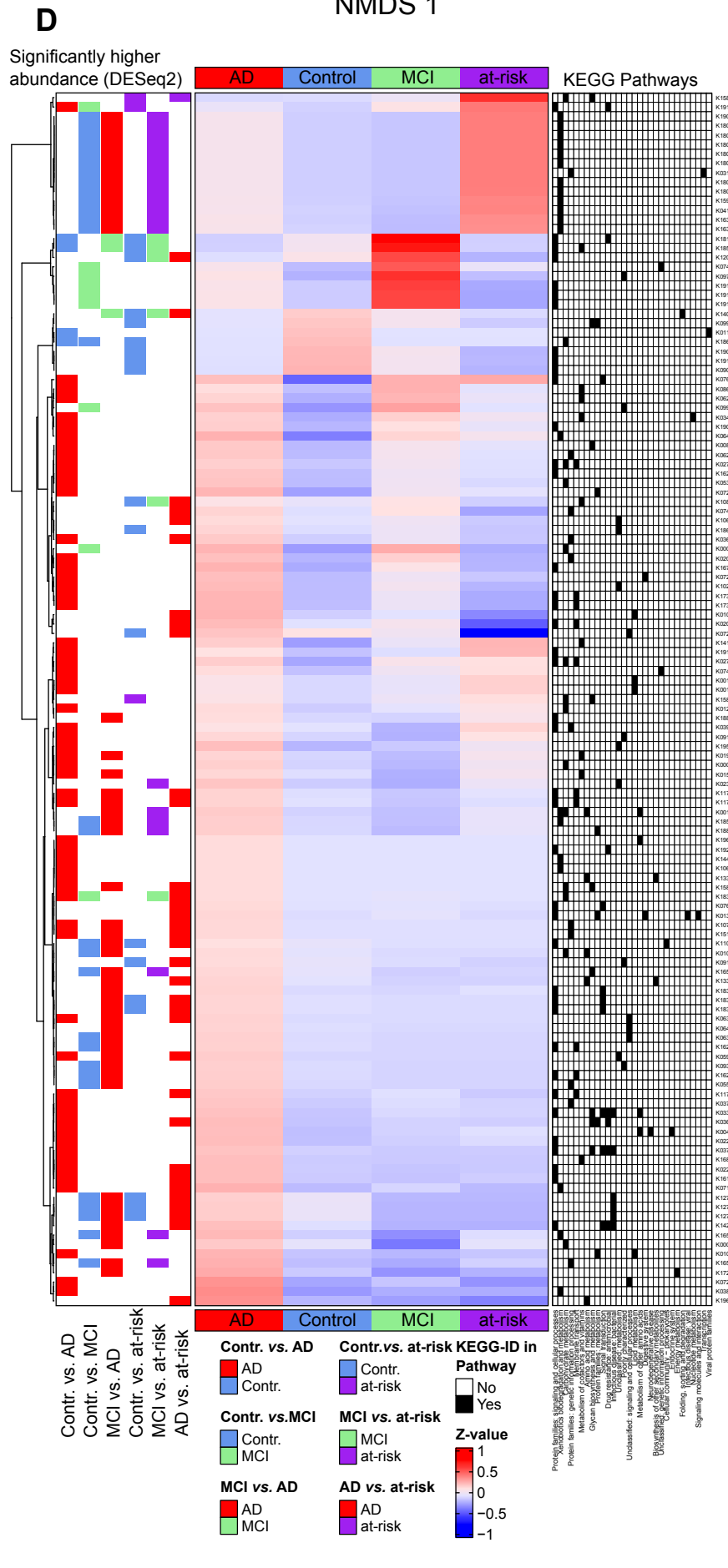

Supplement: pgad427_Supplementary_Data [file pgad427_supplementary_data.zip › PNASNEXUS-PNASNEXUS-2023-00320RR-s07.pdf]

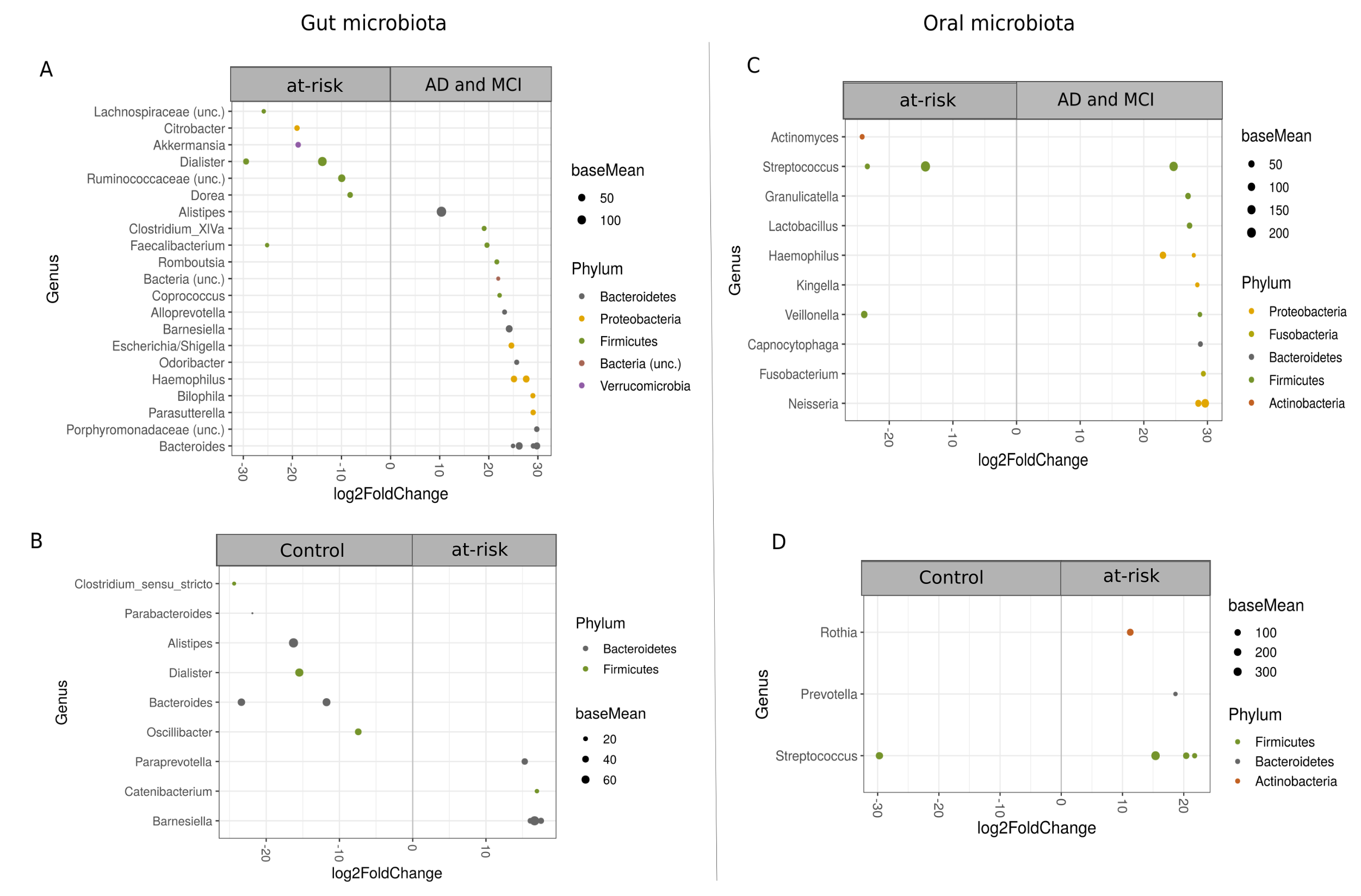

Supplement: pgad427_Supplementary_Data [file pgad427_supplementary_data.zip › PNASNEXUS-PNASNEXUS-2023-00320RR-s08.tif]

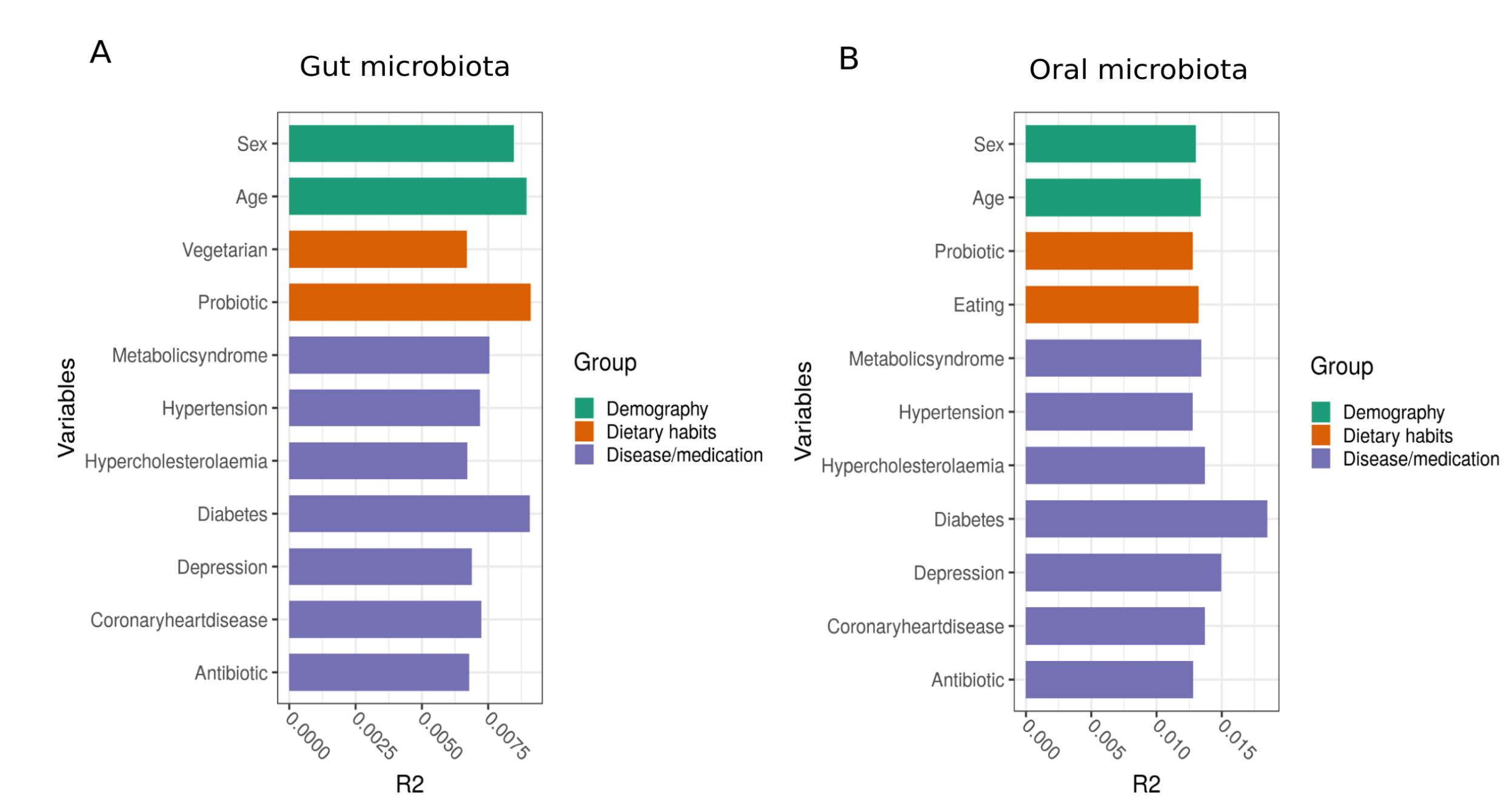

Supplement: pgad427_Supplementary_Data [file pgad427_supplementary_data.zip › PNASNEXUS-PNASNEXUS-2023-00320RR-s10.tif]
